# Post-transcriptional regulation of BRG1 by FIRΔexon2 in gastric cancer

**DOI:** 10.1038/s41389-020-0205-4

**Published:** 2020-02-18

**Authors:** Guzhanuer Ailiken, Kouichi Kitamura, Tyuji Hoshino, Mamoru Satoh, Nobuko Tanaka, Toshinari Minamoto, Bahityar Rahmutulla, Sohei Kobayashi, Masayuki Kano, Tomoaki Tanaka, Atsushi Kaneda, Fumio Nomura, Hisahiro Matsubara, Kazuyuki Matsushita

**Affiliations:** 1 Department of Molecular Diagnosis, Graduate School of Medicine, Chiba University, Chiba, Japan; 2 Department of Laboratory Medicine & Division of Clinical Genetics and Proteomics, Chiba University Hospital, Chiba, Japan; 3 Department of Physical Chemistry, Graduate School of Pharmaceutical Sciences, Chiba University, Chiba, Japan; 4 Divisions of Clinical Mass Spectrometry and Clinical Genetics, Chiba University Hospital, Chiba, Japan; 5 Division of Translational and Clinical Oncology, Cancer Research Institute, Kanazawa University, Kanazawa, Japan; 6 Department of Molecular Oncology, Graduate School of Medicine, Chiba University, Chiba, Japan; 7 Department of Frontier Surgery, Graduate School of Medicine, Chiba University, Chiba, Japan

**Keywords:** High-throughput screening, Cancer genomics, Focal adhesion, Cell signalling, High-throughput screening

## Abstract

Brahma-related gene 1 (BRG1), an ATPase subunit of the SWItch/sucrose non-fermentable (SWI/SNF) chromatin remodeling complex controls multipotent neural crest formation by regulating epithelial-mesenchymal transition (EMT)-related genes with adenosine triphosphate-dependent chromodomain-helicase DNA-binding protein 7 (CHD7). The expression of BRG1 engages in pre-mRNA splicing through interacting RNPs in cancers; however, the detailed molecular pathology of how BRG1and CHD7 relate to cancer development remains largely unveiled. This study demonstrated novel post-transcriptional regulation of BRG1 in EMT and relationship with FIRΔexon2, which is a splicing variant of the far-upstream element-binding protein (FUBP) 1-interacting repressor (FIR) lacking exon 2, which fails to repress *c-myc* transcription in cancers. Previously, we have reported that FIR complete knockout mice (*FIR*^−/−^) was embryonic lethal before E9.5, suggesting FIR is crucial for development. FIRΔexon2 acetylated H3K27 on promoter of BRG1 by CHIP-sequence and suppressed BRG1 expression post-transcriptionally; herein BRG1 suppressed Snai1 that is a transcriptional suppressor of E-cadherin that prevents cancer invasion and metastasis. Ribosomal proteins, hnRNPs, splicing-related factors, poly (A) binding proteins, mRNA-binding proteins, tRNA, DEAD box, and WD-repeat proteins were identified as co-immunoprecipitated proteins with FIR and FIRΔexon2 by redoing exhaustive mass spectrometry analysis. Furthermore, the effect of FIRΔexon2 on *FGF8* mRNA splicing was examined as an indicator of neural development due to impaired CHD7 revealed in CHARGE syndrome. Expectedly, siRNA of FIRΔexon2 altered *FGF8* pre-mRNA splicing, indicated close molecular interaction among FIRΔexon2, BRG1 and CHD7. FIRΔexon2 mRNA was elevated in human gastric cancers but not in non-invasive gastric tumors in *FIR*^*+/*^ mice (K19-Wnt1/C2mE x *FIR*^*+/−*^). The levels of FIR family (FIR, FIRΔexon2 and PUF60), BRG1, Snai1, FBW7, E-cadherin, c-Myc, cyclin-E, and SAP155 increased in the gastric tumors in *FIR*^*+/−*^ mice compared to those expressed in wild-type mice. FIR family, Snai1, cyclin-E, BRG1, and c-Myc showed trends toward higher expression in larger tumors than in smaller tumors in Gan-mice (K19-Wnt1/C2mE). The expressions of BRG1 and Snai1 were positively correlated in the gastric tumors of the Gan-mice. Finally, BRG1 is a candidate substrate of F-box and WD-repeat domain-containing 7 (FBW7) revealed by three-dimensional crystal structure analysis that the U2AF-homology motif (UHM) of FIRΔexon2 interacted with tryptophan-425 and asparate-399 (WD)-like motif in the degron pocket of FBW7 as a UHM-ligand motif. Together, FIRΔexon2 engages in multi-step post-transcriptional regulation of BRG1, affecting EMT through the BRG1/Snai1/E-cadherin pathway and promoting tumor proliferation and invasion of gastric cancers.

## Introduction

The brahma-related gene 1 (BRG1), an ATPase subunit of the SWItch/sucrose non-fermentable (SWI/SNF) chromatin remodeling complex, controls multipotent neural crest formation by regulating epithelial-mesenchymal transition (EMT)-related genes with adenosine triphosphate-dependent chromodomain-helicase DNA-binding protein 7 (CHD7)^1,2^. Integrative analysis identifies co-dependent gene expression regulation of BRG1 and CHD7 at distal regulatory sites in embryonic stem cells^2^. The expression of BRG1 engages in pre-mRNA splicing through interacting RNPs in cancers;^3–5^ however, the detailed molecular pathology of how BRG1and CHD7 relate to cancer development remains largely unknown.

The far-upstream element (FUSE) is a sequence required for the proper expression of the human *c-myc* gene^6^. Yeast two-hybrid analysis revealed that FUBP1 binds to a protein that has transcriptional inhibitory activity, termed FUBP1-interacting repressor (FIR)^7^. *FIR* is a splicing variant lacking exon 5 of poly (U)-binding-splicing factor (*PUF60*)^8^. FIRΔexon2, a splicing variant of FIR that lacks exon 2, failed to repress *c-myc* as a dominant-negative form of FIR in cancers^7^. This study examined a novel mechanism how FIRΔexon2 regulates BRG1 through the epigenome, transcription and alternative splicing in cancer development. To explore this purpose, following functional analyses of FIR and FIRΔexon2 were performed with several cancer cell lines and animal models having non-invasive gastric tumor. First, FIR and FIRΔexon2-binding proteins were revaluated among the data previously identified by exhaustive mass spectrometry analysis^9^. In fact, an autoantibody against FIRΔexon2 was detected in the sera of various cancer patients^10,11^, showing that FIRΔexon2 protein authentically expresses in cancers. Given that c-Myc activates ribosome protein synthesis, FIRΔexon2 is crucial for carcinogenesis in terms of ribosome protein synthesis in cancers. Second, the effect of FIRΔexon2 on *FGF8* mRNA splicing was examined as an indicator of neural development in CHARGE syndrome which is an autosomal-dominant, multiple congenital anomaly condition that is characterized by vision and hearing loss, congenital heart disease, and malformations of craniofacial and other structures. The *PUF60* gene, as well as *CHD7*, is considered responsible for CHARGE syndrome, which cooperatively translocates nucleosomes to permit transcription of *FGF8* by RNA pol II in neural development^12^. Further, the U2AF-homology motif (UHM) of PUF60 has been reported to interact with the WD-repeat of SAP155 (SF3B1)^13^. Additionally, expression of E-cadherin (encoded by the *CDH1*/*Cadherin 1* gene) prevents cancer invasion and metastasis^14,15^. E-cadherin suppresses initiation and epithelial-mesenchymal transition (EMT) in early-stage gastric carcinogenesis^16,17^. Previous studies indicated that loss of F-box and WD-repeat domain-containing 7 (FBW7) induced EMT in cancers^18,19^. The Snai1 transcriptionally represses E-cadherin and promotes tumor proliferation and invasion^20,21^. The regulation of BRG1 by FBW7 has been studied that BRG1 is a substrate of FBW7 and was found to suppress E-cadherin through the FBW7/BRG1/Snai1 axis in gastric cancer^4^. Since FIRΔexon2 was co-immunoprecipitated with WD-repeat proteins^9^, a potential interaction was assessed between FIRΔexon2 and the WD-like motif in the degron pocket of FBW7 by three-dimension crystal analysis^22,23^. Finally, low molecular weight chemicals that bind to FIRΔexon2 were identified from natural chemical libraries (RIKEN, Wako, Saitama, Japan) and we investigated their effect on BRG1/Snai1 pathway for clinical applications. Collectively, this study proposed that FIRΔexon2 engages in multi-step post-transcriptional regulation of BRG1, affecting EMT through the BRG1/Snai1/E-cadherin pathway and promoting tumor proliferation and invasion of gastric cancers.

## Results

### Ribosomal proteins and splicing factors were co-immunoprecipitated with FIR and FIRΔexon2

**Fig. 1:**
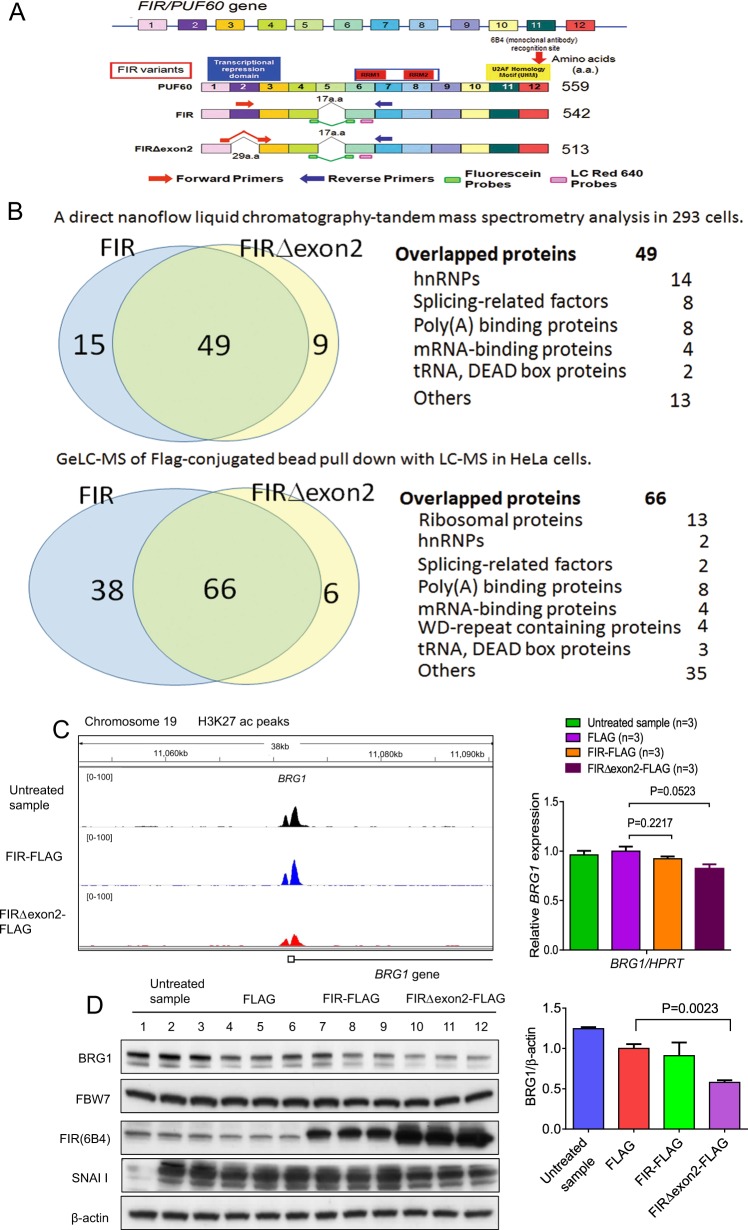
FIRΔexon2, but not FIR, reduced the level of H3K27ac of *BRG1*. **a** Genome structure of PUF60, FIR, and FIRΔexon2 are indicated. The *FIR/PUF60* gene is located at 8q24.3 and contains 12 exons. Primers and probes used for RT-PCR are indicated. PUF60 consists of 559 amino acids (a.a.) and FIR, lacking exon5, is 542 a.a. FIRΔexon2 lacking exon2 of transcriptional repression domain, is 513 a.a. RNA recognition motif and U2AF-homology motif (UHM) are indicated. The 6B4 (Supplementary Table S5) monoclonal antibody recognizes the amino-terminus of FIR family (arrow). FIR family, consisting of FIR, PUF60 and FIRΔexon2, could not be identified separately by the monoclonal antibody (6B4) used in this study. The list of co-immunoprecipitated proteins with FIR and FIRΔexon2 detected by a direct nanoflow liquid chromatography-tandem mass spectrometry analysis in 293 T cells and GeLC-MS of Flag-conjugated bead pull down with LC-MS in HeLa cells (**b**), **c** The overexpression of FIRΔexon2, dominant-negative of FIR, reduced the level of H3K27ac by 37% as compared to that in untreated sample in the *BRG1* genome region, but its mRNA level remained unchanged. One possibility is that translational processes were interfered by FIRΔexon2 and its counterparts. **d** The protein expression of BRG1 was decreased by FIRΔexon2 overexpression.

The *FIR/PUF60* gene is located at 8q24.3 and contains 12 exons. Genomic structure of PUF60, FIR, and FIRΔexon2 are indicated (Fig. 1a). This study revaluated the co-immunoprecipitated proteins with FIR or FIRΔexon2 previously identified by a direct nanoflow liquid chromatography-tandem mass spectrometry analysis of the 293 T cells (Fig. 1b, top**)** or by GeLC-MS of Flag-conjugated bead pull down with LC-MS in HeLa cells (Fig. 1b, botom) according to the previously established cell culture systems^9^. Particularly, ribosomal proteins, hnRNPs, splicing-related factors, poly(A) binding proteins, mRNA-binding proteins, tRNA, WD-repeat proteins or DEAD box proteins were commonly co-immunoprecipitated with FIR or FIRΔexon2 (Table 1 and Supplementary Table S1), indicating that both FIR and FIRΔexon2 participate in post-transcriptional or translational processes. Consequently, FIR families, PUF60, FIR, and FIRΔexon2, potentially link among epigenetic modification, transcription, post-transcription, and alternative splicing in gene regulation. Recently, the ribosomal RPL10 R98S mutation was associated with T-cell type acute lymphocytic leukemia (T-ALL) pathogenesis^24–26^. Notably, *FIR*^+/^^−^*TP53*^−/−^ generated T-ALL^27^, indicating that FIRΔexon2 modifies ribosomal protein synthesis in cancers. Moreover, transformation/transcription domain-associated protein (TRRAP) that is pivotal for rDNA transcription^28^ was co-immunoprecipitated with FIRΔexon2, but not with FIR^9^. Recently, the BRG1/Snai1 pathway has been reported to regulate E-cadherin in gastric cancers^4^, the effect of FIRΔexon2 on the BRG1/Snai1 pathway was investigated in this study. Comprehensive RNA-sequencing and ChIP-sequencing analysis with FIR and FIRΔexon2 overexpressed in HeLa cells were examined to reveal the mechanism. As result, FIRΔexon2, but not FIR, reduced the level of H3K27ac at the *BRG1* promotor (Fig. 1c). Notably, the overexpression of FIRΔexon2 reduced the level of H3K27ac by 37% compared to that in the untreated sample in the *BRG1* genome region, but its mRNA level remained unchanged (Fig. 1c). The protein expression of BRG1 was decreased by FIRΔexon2 overexpression (Fig. 1d), suggesting that FIRΔexon2 partly affects nucleosome remodeling. Possibly, the histone modification of translational processes in BRG1 expression were interrupted by FIRΔexon2 and its complex.

**Table 1:** List of FIR or FIRΔexon2 binding proteins detected by GeLC-MS of Flag-conjugated beads pull down with LC-MS in HeLa cells.

	IPI	kDa	Unique peptide	Unique peptide
Identified protein name	Accession number	Molecular weight (kDa)	FIR_FlagIP	FIRΔexon2_FlagIP
Isoform 1 of Poly(U)-binding-splicing factor PUF60	IPI00069750	60	5	10
Actin, alpha cardiac muscle 1	IPI00023006	42	5	7
LIM domain and actin-binding 1 isoform a	IPI00883896	85	13	6
60 kDa heat shock protein, mitochondrial	IPI00784154	61	4	5
Isoform 1 of U5 small nuclear ribonucleoprotein 200 kDa helicase	IPI00420014	245	1	5
Actin-related protein 3	IPI00028091	47	4	4
Isoform 2 of Suppressor of SWI4 1 homolog	IPI00219793	52	3	4
Isoform 1 of Drebrin	IPI00003406	71	7	3
60 S ribosomal protein L18	IPI00215719	22	5	3
ATP-dependent RNA helicase DDX50	IPI00031554	83	4	3
WD-repeat-containing protein 3	IPI00009471	106	3	3
Isoform 1 of RNA-binding protein 39	IPI00163505	59	3	3
RNA-binding protein 28	IPI00304187	86	2	3
WD-repeat-containing protein 36	IPI00169325	105	1	3
WD-repeat-containing protein 75	IPI00217240	95	1	3
cDNA FLJ56443, highly similar to Putative ATP-dependent RNA helicase DHX33	IPI00302860	85	1	3
Glioma tumor suppressor candidate region gene 2 protein	IPI00024567	54	1	3
CCAAT/enhancer-binding protein zeta	IPI00306723	121	1	3
Protein MAK16 homolog	IPI00332428	35	1	3
Actin-related protein 2/3 complex subunit 2	IPI00005161	34	6	2
Isoform 1 of Guanine nucleotide-binding protein G(i), alpha-2 subunit	IPI00748145	40	3	2
F-actin-capping protein subunit alpha-2	IPI00026182	33	3	2
Poly [ADP-ribose] polymerase 1	IPI00449049	113	2	2
ATP-dependent RNA helicase DDX24	IPI00006987	96	2	2
Isoform 1 of Transformer-2 protein homolog beta	IPI00301503	34	2	2
RNA-binding protein, autoantigenic (HnRNP-associated with lethal yellow homolog (Mouse)), isoform CRA_a (Fragment)	IPI00011268	33	2	2
Apolipoprotein A-I	IPI00021841	31	2	2
40 S ribosomal protein S2	IPI00013485	31	2	2
60 S ribosomal protein L10a	IPI00412579	25	2	2
60 S ribosomal protein L15	IPI00470528	24	2	2
40 S ribosomal protein S15	IPI00479058	17	2	2
WD-repeat-containing protein 43	IPI00055954	79	1	2
Isoform 1 of Fragile X mental retardation syndrome-related protein 1	IPI00016249	70	1	2
Isoform 8 of Fragile X mental retardation 1 protein	IPI00645666	66	1	2
60 S ribosomal protein L21	IPI00247583	19	1	2
Thyroid hormone receptor-associated protein 3	IPI00104050	109	1	2
Fragile X mental retardation syndrome-related protein 2	IPI00016250	77	1	2
TDP43	IPI00025815	45	1	2
Forty-two-three domain-containing protein 1	IPI00289907	36	1	2
Isoform 1 of Uncharacterized protein C1orf77	IPI00300990	26	1	2
40 S ribosomal protein S14	IPI00026271	16	1	2
Heat shock protein HSP 90-beta	IPI00414676	83	8	1
Actin-related protein 2	IPI00005159	45	7	1
Uncharacterized protein C19orf21	IPI00217121	75	6	1
Isoform 4 of Myosin-XIX	IPI00062809	87	4	1
myosin VA isoform 2	IPI00873959	212	4	1
Splicing factor 3B subunit 1(SAP155)	IPI00026089	146	4	1
Ribosome biogenesis protein BOP1	IPI00028955	84	3	1
60 S ribosomal protein L34	IPI00219160	13	3	1
Actin-related protein 2/3 complex subunit 1B	IPI00005160	41	3	1
protein phosphatase 1, catalytic subunit, alpha isoform 3	IPI00027423	39	3	1
40 S ribosomal protein S16	IPI00221092	16	3	1
Actin-related protein 2/3 complex subunit 1 A	IPI00333068	42	2	1
Isoform 1 of 60 S ribosomal protein L11	IPI00376798	20	2	1
Histone H2A.V	IPI00018278	14	2	1
Enhancer of rudimentary homolog	IPI00029631	12	2	1
Isoform 1 of DNA-dependent protein kinase catalytic subunit	IPI00296337	469	2	1
protein phosphatase 1, regulatory subunit 9B	IPI00045550	89	2	1
cDNA FLJ37875 fis, clone BRSSN2018771, highly similar to Poly(A)-binding protein 1	IPI00796945	71	2	1
Isoform A of Phosphate carrier protein, mitochondrial	IPI00022202	40	2	1
Isoform Long of Transformer-2 protein homolog alpha	IPI00013891	33	2	1
similar to beta-actin	IPI00739464	17	2	1
Isoform 1 of Synaptic glycoprotein SC2	IPI00100656	36	2	1
ADP/ATP translocase 3	IPI00291467	33	2	1
60 S ribosomal protein L19	IPI00025329	23	2	1
Cystatin-A	IPI00032325	11	2	1
Isoform 1 of Spectrin beta chain, brain 2	IPI00012645	271	16	0
epiplakin 1	IPI00010951	556	12	0
Src substrate cortactin	IPI00029601	62	11	0
Isoform 1 of Myosin-XVIIIa	IPI00760846	233	8	0
Isoform 2 of Myosin-VI	IPI00008455	146	6	0
Pre-mRNA-processing-splicing factor 8	IPI00007928	274	5	0
Isoform 2 of Supervillin	IPI00018370	201	5	0
Isoform 1 of Actin-binding LIM protein 1	IPI00329495	88	5	0
Tropomodulin-1	IPI00002375	41	5	0
Isoform 1 of Probable ATP-dependent RNA helicase DDX31	IPI00043990	94	4	0
40 S ribosomal protein S3	IPI00011253	27	4	0
Myosin-Ie	IPI00329672	127	3	0
Isoform 1 of Nexilin	IPI00180404	81	3	0
D-3-phosphoglycerate dehydrogenase	IPI00011200	57	3	0
EF-hand domain-containing protein D1	IPI00031091	27	3	0
Cofilin-1	IPI00012011	19	3	0
Filamin A	IPI00909642	246	2	0
Isoform 2 of Nuclear mitotic apparatus protein 1	IPI00006196	237	2	0
Isoform 1 of Chromodomain-helicase-DNA-binding protein 4	IPI00000846	218	2	0
Importin subunit beta-1	IPI00001639	97	2	0
Nuclear cap-binding protein subunit 1	IPI00019380	92	2	0
ATP-dependent RNA helicase DDX51	IPI00217541	72	2	0
Plastin-3	IPI00216694	71	2	0
Isoform 1 of Heterogeneous nuclear ribonucleoprotein Q	IPI00018140	70	2	0
cDNA FLJ41552 fis, clone COLON2004478, highly similar to Protein Tro alpha1 H,myeloma	IPI00647704	53	2	0
Pyruvate kinase	IPI00847989	50	2	0
Isoform 1 of Sequestosome-1	IPI00179473	48	2	0
Tricarboxylate transport protein, mitochondrial	IPI00294159	34	2	0
60 S ribosomal protein L6	IPI00329389	33	2	0
Isoform 2 of Voltage-dependent anion-selective channel protein 2	IPI00024145	30	2	0
Metaxin-2	IPI00025717	30	2	0
60 S ribosomal protein L7-like 1	IPI00456940	29	2	0
14-3-3 protein epsilon	IPI00000816	29	2	0
Isoform 1 of Splicing factor, arginine/serine-rich 7	IPI00003377	27	2	0
Chloride intracellular channel protein 1	IPI00010896	27	2	0
Isoform 1 of 60 S ribosome subunit biogenesis protein NIP7 homolog	IPI00007175	20	2	0
Profilin-1	IPI00216691	15	2	0
Ribosomal protein 26 (RPS26) pseudogene	IPI00401819	13	2	0
Periodic tryptophan protein 2 homolog	IPI00300078	102	0	3
Isoform 1 of Transformation/transcription domain-associated protein	IPI00069084	438	0	2
hypothetical LOC731605	IPI00886987	100	0	2
DEAD (Asp-Glu-Ala-Asp) box polypeptide 54 isoform 1	IPI00152510	99	0	2
Isoform Long of Splicing factor, proline- and glutamine-rich	IPI00010740	76	0	2
Ribosome biogenesis protein WDR12	IPI00304232	48	0	2

### *FGF8* splicing variant analysis by up- or downregulated FIR or FIRΔexon2 in cancer cells

**Fig. 2:**
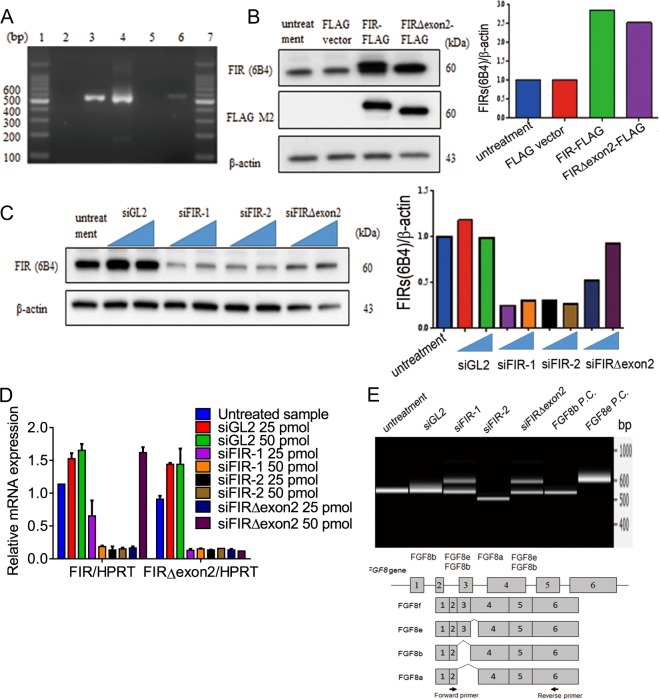
FIR and FIRΔexon2 affected alternative splicing of *FGF8* mRNA. **a** Expression of *FGF8* analysis was confirmed by PCR using cancer cell lines, HeLa, MCF-7 and Jurkat cells. Lane 1 and 7: 100 bp marker, lane 2: HeLa cells, lane 3: MCF-7 cells, lane 4: Jurkat cells, lane 5: negative control, lane 6: FGF8 positive control. **b** MCF-7 cells were transfected with FIR-FLAG, FIRΔexon2-FLAG (left). Bar graph was showed the relative intensity of bands (right). The internal control is β-actin. **c** Western blotting analysis of knockdown of FIR or FIRΔexon2 in MCF-7 cells (left). Bar graph is normalized intensity of bands (right). Internal control protein is β-actin. siRNA concentration of FIR-1, FIR-2 and FIRΔexon2 is 25 and 50 pmol respectively. **d** qRT-PCR of FIR, FIRΔexon2 mRNAs. siGL2 is control siRNA. Internal control gene is *HPRT*. **e** Splicing variants analysis of *FGF8* using bioanalyzer electrophoresis in MCF-7 cells. siRNA concentration of FIR-1, FIR-2 and FIRΔexon2 was 50 pmol.

FIR complete knockout C57BL/6 mice (*FIR*^−/−^) was embryonic lethal before E9.5; strongly suggests that FIR is crucial for development^27^. Recently, the germ line mutations or deletions in the RNA recognition motifs (RRMs) of the *PUF60* gene were reported in Verheij or CHARGE syndrome^12,29–31^. In CHARGE syndrome patients, protein interactions are spoiled in alternative splicing of *FGF8* mRNA pre-mRNA processing in neural development due to dysfunction among BRG1, SWI/SNF and CHD7. Furthermore, CHD7 binds to unmethylated active rDNA and promotes rRNA transcription^32^. Five gastric cancer cell lines, MNK7, MNK45, MNK74, NUGC3, and NIGC4, were examined FGF8 expression at protein and mRNA levels (Supplementary Figs. S1a, S1b). NUGC4 showed highest expression of FGF8 expression among them, however, the transfection efficiency of FIR-FLAG and FIRΔexon2-FLAG plasmids were low in NUGC4, adherent round cells (Supplementary Fig. S1c), or Jurkat cells; therefore, MCF7 were used in this study^33^. Expectedly, FIR or FIRΔexon2 affected to the alternative splicing of *FGF8* mRNA expression revealed by PCR analysis in cancer cell lines, MCF-7, HeLa, and Jurkat cells (Lanes 1 and 7; 100 bp marker, lane 2: HeLa cells, lane 3; MCF-7 cells, lane 4; Jurkat cells, lane 5; negative control, lane 6; FGF8 positive control; Fig. 2a). The internal control was β-actin (Fig. 2b). Western blotting analysis of knockdown of FIR or FIRΔexon2 in MCF-7 cells and normalized intensity of bands were indicated (Fig. 2c). qRT-PCR of *FIR* and *FIRΔexon2* mRNAs are shown in Fig. 2d. Overexpression or knockdown of FIR or FIRΔexon2 changed the alternative splicing of *FGF8* pre-mRNA in MCF-7 cells (Fig. 2e). These results indicated that FIR or FIRΔexon2 surely engages in the alternative splicing process of *FGF8* pre-mRNA at least in cancer cells, in vitro.

### Elevated expression of BRG1 and FIR, but not FIRΔexon2, in Gan-mice as a non-invasive early gastric cancer model

**Fig. 3:**
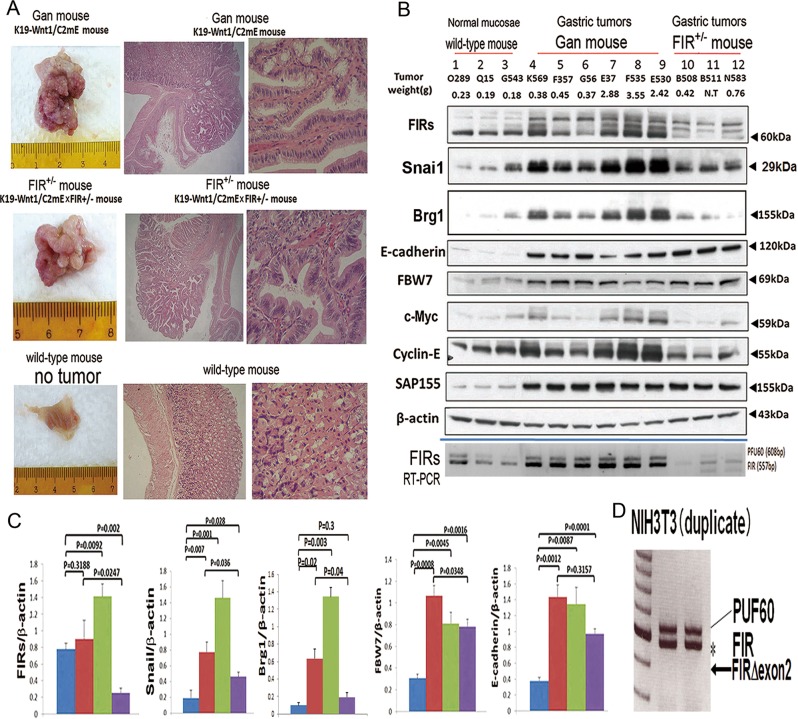
Tumorigenesis and histological type of Gan-mouse and *FIR*^*+/−*^ mouse. **a**, **top panels** Picture of Gan-mouse gastric tumor and *FIR*^*+/*^^−^ mouse gastric tumor. **a**, **middle panels** hematoxylin-eosin staining photo (40 × ) of Gan-mouse and *FIR*^*+/*^^−^ mouse gastric tumor. Note, lamina muscularis mucosae was intact (**a**, **top and middle panels**). **a**, **bottom panels** Wild-type mice showed no gastric tumors during the period of this experiment. Normal gastric mucosa was used as a negative control. **b** Expression of FIR and related proteins was examined by western blotting in wild-type mice tissues, as well as in Gan-mouse and *FIR*^*+/*^^−^ mice gastric tumor tissues. The expressions of FIR family, Snai1, BRG1, E-cadherin, FBW7, c-Myc, cyclin-E, SAP155, and hnRNPA1 of wild-type, Gan-mice, and *FIR*^*+/−*^ mice were examined. Frozen tissues samples of mice gastric tumors were obtained and proteins expression profiles examined by western blotting in three different genotypes: wild-type mouse (lanes 1–3), Gan-mice (gastric tumor wet weight < 0.5 g) (lanes 4–6), Gan-mice (gastric tumor wet weight > 2.0 g) (lanes 7–9) and *FIR*^*+/−*^ mice (lanes 10–12). **c** The extent of the signals detected by western blotting revealed in Fig. 1 was quantified by densitometry. **d** Alternative splicing forms of PUF60 and FIR mRNAs were detected in NIH3T3 cells by reverse-transcription-polymerase chain reaction (RT-PCR).

To study the effect of FIRΔexon2 on BRG1 expression in tumor development, Gan-mice (K19-Wnt1/C2mE) that develop non-invasive gastric tumors at a frequency of 100% were examined^34^. They are considered as a model of non-invasive gastric cancer (Fig. 3a, top panels). Gan x *FIR*^*+/*^^−^ mice (K19-Wnt1/C2mE x *FIR*^*+/*^^−^ mice) were prepared to employ an invasive gastric tumor model; however, the gastric tumors remained non-invasive (Fig. 3a, middle panels). No wild-type mouse developed a gastric tumor during the period of observation (Fig. 3a, bottom panels, Supplementary Table S2). Additionally, there was no apparent difference in tumor growth speed and histological type between the Gan and *FIR*^*+/−*^ mice (data not shown). Expression profiles of the FIR or FIRΔexon2-related proteins^9^ were examined in gastric tumors of Gan and *FIR*^*+/−*^ mice. The levels of FIR family, BRG1, Snai1, FBW7, E-cadherin, c-Myc, cyclin-E, and SAP155 increased in the gastric tumors in *FIR*^*+/−*^ mice compared to those expressed in wild-type mice. Interestingly, FIR family, Snai1, cyclin-E, BRG1, and c-Myc showed trends toward higher expression in larger tumors (Fig. 3b, lanes 7–9) than in smaller tumors (Fig. 3b, lanes 4–6) in Gan-mice. An alternative splicing variant forms of FIR, FIRΔexon2, were indicated by qRT-PCR (Fig. 3b, bottom). In contrast, the expressions of cyclin-E, Snai1, and BRG1 were decreased, whereas FBW7 and E-cadherin expressions were sustained in *FIR*^*+/−*^ mice tumors. Although the *TP53* gene mutation affects the expression of various cellular proteins, the *TP53* gene mutation status did not significantly affect the expression levels of FIR, FBW7, and SAP155 in esophageal cancer cells^23^. The expression levels of proteins were quantified by densitometry (Fig. 3c), and the statistical significance of these protein expressions were confirmed (Fig. 3c and Supplementary Fig. S2a). In mice fibroblast NIH3T3 cells, FIR and PUF60 mRNAs were detected, but FIRΔexon2 mRNA was not detected by RT-PCR (Fig. 3d). The expressions of BRG1 and Snai1 were positively correlated in the gastric tumors of the Gan-mice (Supplementary Fig. S2b). The epitope of the anti-FIR monoclonal antibody, 6B4, is located in the UHM at the carboxyl terminus (a thick arrow, Fig. 1a) in FIR, PUF60, and FIRΔexon2^35^.

### Expression of BRG1, FIR and FIRΔexon2 in human invasive gastric cancers

**Fig. 4:**
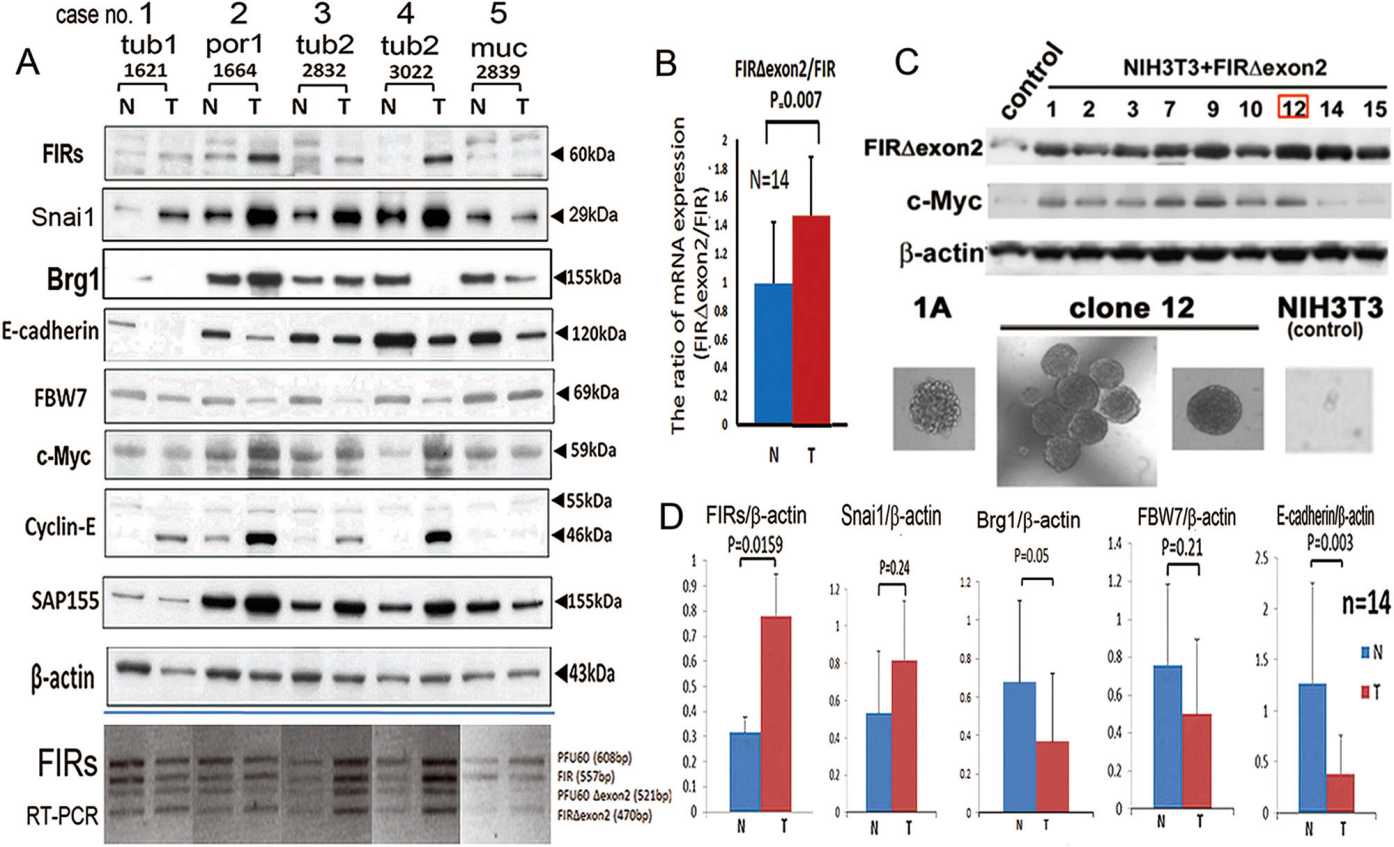
Protein expression profiles of human gastric cancer tissue samples. **a** Expressions of FIR family and related proteins were examined by western blotting in five paired-tumor (T) and adjacent non-tumor (N) tissue samples from human gastric cancer tissues. **a**, **bottom** Four splicing variants of the *FIR/PUF60* gene PUF60, FIR, PUF60Δexon2, and FIRΔexon2 were detected in gastric cancer tissues in five paired tumors (T) and adjacent non-tumors (N). tub1: well-differentiated tubular adenocarcinoma, tub2: moderately differentiated tubular adenocarcinoma; por1: poorly differentiated adenocarcinoma, solid type; muc: mucinous adenocarcinoma. **b** The ratio of mRNA expression of FIRΔexon2/FIR was significantly higher in gastric cancer tissues (T) that in corresponding non-cancer tissues (N). *N* = 14, *P* = 0.007 by Student’s *t*-test. **c** Clones of stably transfected pcDNA3.1-FIRΔexon2 plasmids (clones 1,2,3,7,9,10,12,14, and 15 among 30 clones). At least 30 clones were screened by immunoblotting and immunostaining with anti-FLAG and anti-FIR antibodies (6B4) to find FIR-FLAG-expressing clones for FIR-FLAG stably expressing cells, or with anti-c-Myc antibody to examine c-Myc expression for FIRΔexon2 stably expressing cells. Soft-agar colony formation assay of clone12. The cells of clone12 (2 × 10^3^) were inoculated in 0.3% low-melting-temperature agarose (FMC Bio Products, Rockland, ME, USA) in DMEM supplemented with 10% FCS, and colonies were scored after incubating for 2 weeks. 1 A cells was a positive control cells provided by Dr Ariga^27^. **d** Comparison of protein expressions from five paired tumors determined by statistical analysis in gastric cancer tissues in 14 paired tumors (T) and adjacent non-tumors (N). *N* = 5, *P* < 0.01, R < 1.0 was obtained by Student’s t-test.

Next, the effect of FIRΔexon2 on BRG1 expression in human invasive gastric cancers was examined by western blot analysis for various molecules expressed in the gastric tissues of 14 representative patients (cases no 1–5 in Fig. 4a and cases no 6–14 in Supplementary Fig. S3). Unlike Gan-mice (Fig. S2b), the expressions of BRG1 and Snai1 were not significantly correlated (Supplementary Fig. S4a, b). Snai1 expression was increased but BRG1 was decreased in (T) than those in (N) in human gastric cancer tissues (Supplementary Fig. S4c). Given the FBW7/BRG1 signaling axis governs E-cadherin expression by modulates Snai1 in gastric cancer metastasis^4^, FIRΔexon2 is a candidate that affects FBW7/BRG1 signaling axis. Invasive gastric cancer showed that the expressions of E-cadherin and FBW7 were decreased in human gastric tumor tissues relative to those of their non-tumorous counterparts (Fig. 4a). FIRΔexon2 mRNA was significantly highly expressed in cancers compared to their non-cancerous counterpart tissues (Fig. 4a, bottom panel)^27,35^. The ratio of mRNA expressions of FIRΔexon2/FIR was significantly higher in (T) than in (N) (Fig. 4b). Furthermore, stably transfected pcDNA-3.1/FIRΔexon2 clones of NIH3T3 cells showed colony formation in a soft-agar assay (Fig. 4c). The expressions of FIR family and Snai1 were significantly higher in tumor tissues (T) than in their non-cancerous counterparts (N) (Fig. 4d). Protein expression profiles in Gan-mice and human gastric tumor tissue were indicated (Supplementary Table S3). These results indicated that FIRΔexon2 was expressed in human gastric cancers but not in *FIR*^+/−^ mice, and was engaged in tumor invasive behavior to a certain extent.

### Potential interaction between a novel WD-like motif (W425 and D399) of FBW7 and UHM of FIR protein: a three-dimensional crystal structure analysis

**Fig. 5:**
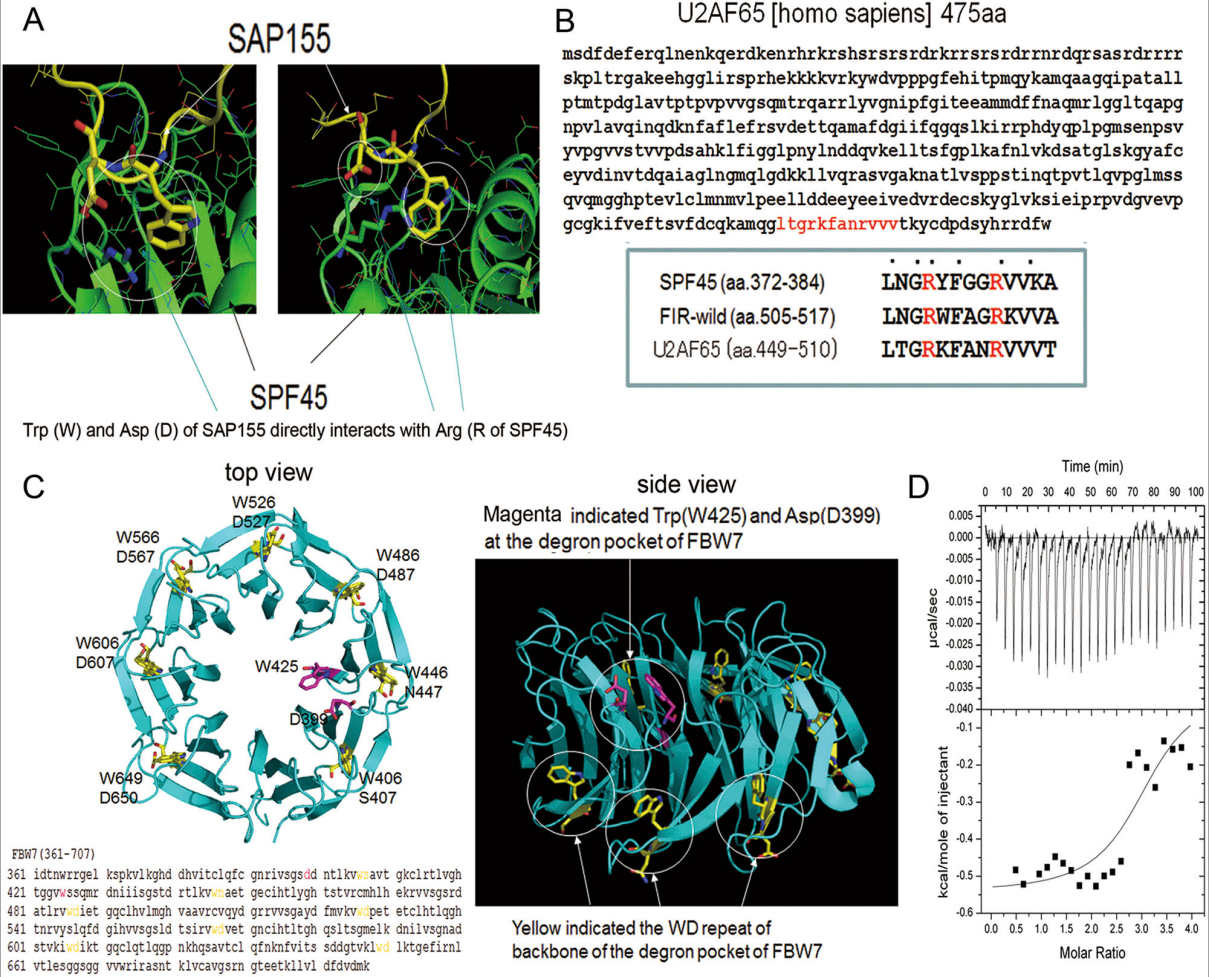
Crystal structure analysis of the interaction between FBW7 and FIR/PUF60-UHM. **a** The amino sequence LNGRYFGGRVVKA in SPF45 is similarto sequences in the C-terminal domains of FIR and U2AF65. A comparison of two crystal structures indicates that the positions and configurations of W425 and D399 in FBW7 are considerably similar to those of SAP155. FBW7 has many WD-motifs, and most of the motifs are involved in the conformational stabilization of the WD-repeated domain (molecules indicated in yellow). The binding structure between SF3B1 and one of the splicing factors containing UHM, SPF45 (human splicing factor 45), has previously been clarified by X-ray crystal analysis (PDB code: #2PEH)^29^. In the 2PEH structure, the crystal unit cell contains two SPF45 recombinant proteins (amino acids: 301–401) and two SF3B1 partialpe ptides (aa: 333–342). **b** Amino acids sequence of U2AF65 (475aa). Similarity among SPF45, FIR, and U2AF65. **c** There is an extra pair of W425 and D399 at the center of the WD-repeated domain (molecules indicated in magenta). Since the binding pocket of FBW7 contains the WD-motif that is expected to interact with FIRΔexon2, the chemical skeleton of the two synthesized compounds is regarded as a WD-mimicking form. A low molecular weight artificial chemical, BK697, that inhibits FIRΔexon2, as shown by in silico analysis, was synthesized. **d** The isothermal titration calorimetry (ITC) measurement of FBW7 with FIRΔexon2 suggested the molecular interaction between two proteins. The exothermic peaks were observed in the initial 17 injections.The peak level was decreased in the later injections. Due to the quick upward change in the ITC thermogram, the association constant is >108 M^−1^. Since the binding reaction is exothermic, the binding of FBW7 and FIRΔexon2 is enthalpically driven.This result is compatible with the importance of Asp of FBW7 in the molecular binding because Asp usually contributes to hydrophilic interactions, such as hydrogen bond formation, between protein molecules.

Finally, possibility of the inhibition of FIRΔexon2 in the BRG1 degradation by FBW7 was investigated^4^. FBW7 is a member of the Skp1-Cull-F-box (SCF) type ubiquitin ligase complex and participates in proteasomal degradation of various tumor-promoting molecules and is considered to be a bona fide tumor suppressor^36^. WD-repeat proteins were co-immunoprecipitated with FIR and FIRΔexon2 (Table 1). If FIR and FIRΔexon2 interact with FBW7, FBW7 need to contain the WD-like domain. In this study, the interaction between UHM of FIR or PUF60 and the WD-like motif (W425 and D399) of FBW7 was found with three-dimensional crystal structure analysis^23^. The FIR family contains the UHM at the carboxyl terminus (Fig. 1a, arrow) that potentially interacts with WD-like motifs, such as the UHM-ligand motif, in the degron pocket of FBW7 (Fig. 5a), and thus might interfere with ubiquitination of its substrate proteins^22,23,37,38^. To research this possibility, the interaction between UHM of FIR or PUF60 and the WD-like motif (W425 and D399) of FBW7 was examined by three-dimensional crystal structure analysis^22^. The binding structure between SF3B1 and one of the splicing factors containing UHM and SPF45 (human splicing factor 45) has previously been clarified by X-ray crystal analysis (PDB code: #2PEH)^13^ (Fig. 5a). In the 2PEH structure, the crystal unit cell contains two SPF45 recombinant proteins (amino acids: 301–401) and two SF3B1 partial peptides (aa: 333–342). The amino acid sequence LNGRYFGGRVVKA in SPF45 (aa: 372–384) is similar to sequences at the C-terminal domains of FIR-wild (aa: 505–517) and U2AF65 (aa: 449–510; Fig. 5b). A comparison of the two crystal structures indicates that the positions and configurations of W425 and D399 in FBW7 are considerably similar to those in SAP155 (Fig. 5c). Therefore, FBW7 possibly binds to FIR in a similar manner as the way SAP155 binds to SPF45. The extra pair of W and D at the WD-repeated domain (molecules indicated in yellow, Fig. 5c -right) cannot be involved in the ligand recognition of the phosphorylated peptides, but the WD pair can interact with the peptide with the above-mentioned conserved sequence based on their structures (molecules indicated in magenta, Fig. 5c -right). This result is compatible with the importance of Asp of FBW7 in the molecular binding because Asp usually contributes to hydrophilic interactions, such as hydrogen bond formation between molecules (Fig. 5d). Therefore, FIRΔexon2 interferes with the WD-like motif of FBW7 and potentially inhibits its function. Thereafter, small molecular weight chemicals were screened that inhibit the FIRΔexon2-FWB7 interaction^23^.

### Decreased E-cadherin expression promoted migration of gastric cancer cells

**Fig. 6:**
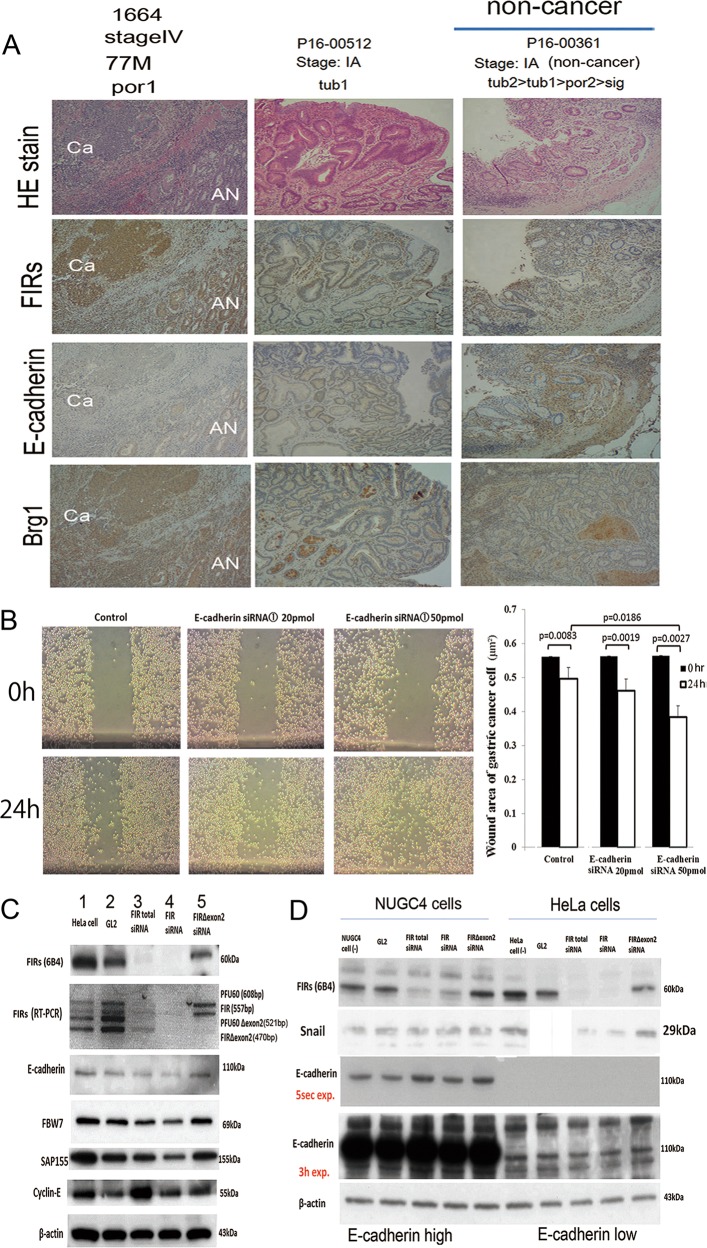
E-cadherin siRNA promotedg astric cancer cells migration in the wound-healing assay. **a** E-cadherin expression decreased, whereas FIR family and BRG1 expressions increased in cancer tissues (Ca) relative to those in adjacent normal tissues revealed by immunohistochemical staining. Case numbers 2832, 1621, and 1664 are listed in Suppl. Table S4. In the non-invasive early stage (IA) of differentiated cancers, the expressions of E-cadherin and FBW7 were decreased. Case number P16-00361 are listed in Suppl. Table S8. **b** Migration of E-cadherin siRNAs transfected into NUGC4 cells and corresponding control cells was measured by wound-healing assay. P < 0.01 was obtained by Student’s t-test. **c** FIR family and related protein expressions were examined aftertreatment of HeLa cells withthe FIRsiRNAs. GL2 siRNA was transfected as the negativecontrol. After 48 h of transfection, whole-cell extracts were analyzed by western blotting. Three types of FIR siRNAs were transfected into HeLa cells. Lane 2 is GL2 siRNA control transfection, lane 3 is 20 pmol of total FIR siRNA transfection, lane 4 is 20 pmol of FIRsiRNA transfection, and lane 5 is 20 pmolof FIRΔexon2 siRNA transfection. **d** E-cadherin expression in HeLa cells. The level of E-cadherin expression was much higher in NUGC4 than that of HeLa cells. Three types of FIR siRNAs were transfected into NUGC4 and HeLa cells. GL2 siRNA is internal control. 20 pmol of total FIR siRNA, 20 pmol of FIR siRNA, and 20 pmol of FIRΔexon2 siRNA transfection were performed. After 48 h of transfection, whole-cell extracts were analyzed by western blotting.

Immunohistochemical staining showed that FIR and FIRΔexon2 (FIR family), Snai1, and BRG1 expressions were increased but FBW7 and E-cadherin expressions were decreased in human gastric cancers (Fig. 6a, Supplementary Figs. S3 and S4a-c); however, the expression of BRG1 was reduced in some cases (cases 1 and 4 in Fig. 2a; cases 6,8,10,12, and 14 in Supplementary Fig. S3). Given that E-cadherin is involved in cell–cell adhesion and the invasion of cancers^39^, knockdown of E-cadherin by siRNA may affect the migration of cancer cells according to an MTS assay in NUGC cells (cell proliferation assay; Fig. 6b). Firefly luciferase gene (GL2) siRNA was transfected as a negative control. As expected, a wound-healing assay showed that the migration of NUGC cells was enhanced by transfection of E-cadherin-specific siRNA (Fig. 6b). SiRNA of FIRΔexon2 was slightly increased but siRNA of FIR decreased E-cadherin expression (Fig. 6c), indicating FIRΔexon2 was decreases E-cadherin expression in HeLa cells. The level of E-cadherin expression was much higher in NUGC4 than that of HeLa cells (Fig. 6d).

### A low molecular weight chemical, BK697, identified as a FIRΔexon2 inhibitor, suppressed tumor growth by decreasing FIR family and E-cadherin and increasing Snai1 expression

**Fig. 7:**
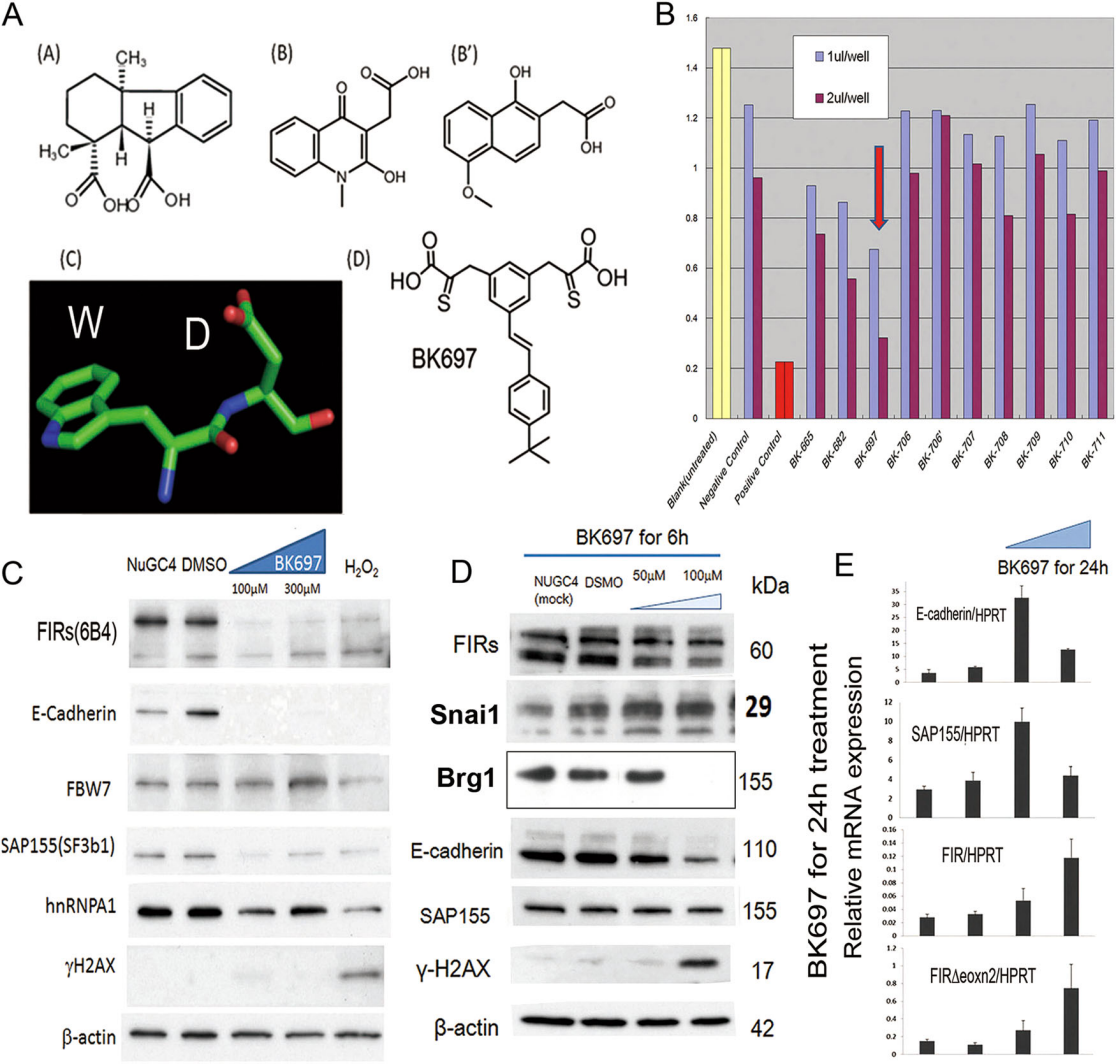
Chemical structure of BK697 and its effect to HeLa and NUGC4 cells. **a** The conformations of the two inhibitory compounds were found to resemble the WD-motif. **b** Among the chemical compounds, BK697 showed significant inhibition of cell growth by MTT assay. Chemicals were diluted in DMSO at a concentration of 10 mM, and we used 1 and 2 μL/well/100 μL medium (final concentrations in the medium were 0.1 mM and 0.2 mM, respectively). The same volume of DMSO was used as a negative control. The same volume of 3% H_2_O_2_ was used as a positive control. Untreated cells were used as the blank. **c**, **d** BK697 suppressed FIR family as well as SAP155 and hnRNPA1 expression in HeLa cells in a dose-dependent manner. **e** FIR and FIRΔexon2 mRNAs were examined by quantitative reverse-transcription-polymerase chain reaction after 24 h treatment of BK697. HPRT mRNA was used as an internal control.

**Fig. 8:**
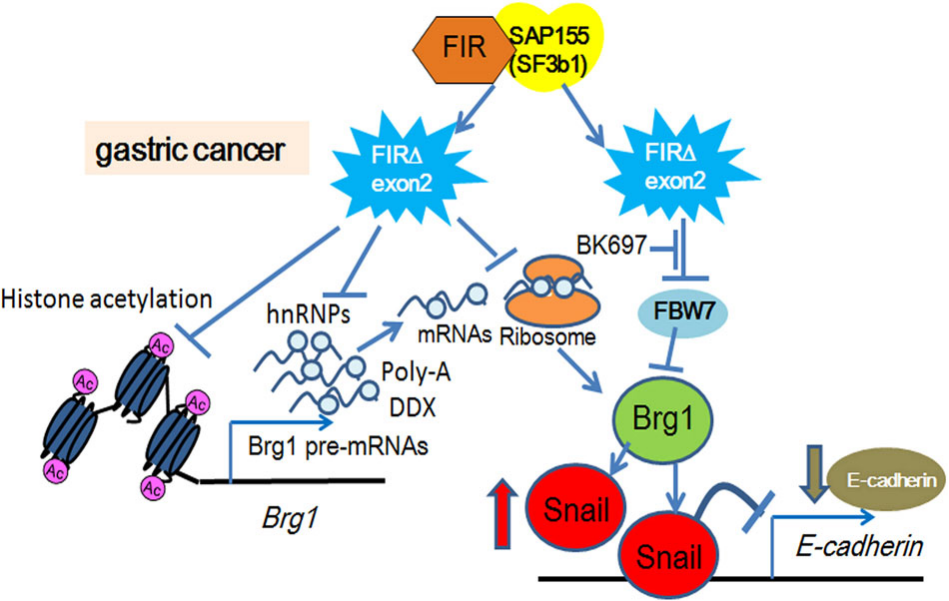
Alternative splicing product of *FIR/PUF60* gene, FIRΔexon2, contributes to E-cadherin suppression through post-transcriptional regulation of BRG1. FIRΔexon2 is an alternative splicing variant form of FIR. FIRΔexon2 is expressed in cancers when FIR pre-mRNA is disturbed by an autocatalytic mechanism through inhibiting the FIR-SAP155 splicing complex^9^. Ribosomal proteins, hnRNPs, splicing-related factors, poly(A) binding proteins, mRNA-binding proteins, tRNA, or DEAD box proteins (DDX) were commonly co-immunoprecipitated with FIR or FIRΔexon2, indicating that both FIR and FIRΔexon2 participate in post-transcriptional or translational processes. FIRΔexon2, but not FIR, reduced the level of H3K27ac at the *BRG1* promotor. The protein expression of BRG1 was decreased by FIRΔexon2 overexpression, suggesting that FIRΔexon2 partly affects nucleosome remodeling. FIRΔexon2 potentially inhibits the accession of substrate proteins of FBW7 to its degron pocket (c.f. Fig. 6). BK697 is a small molecular weight chemical containing a WD-like domain that inhibits FIR/FIRΔexon2. Snai1 is a transcriptional repressor of E-cadherin. Together, the suppression of E-cadherin by BK697 was at least partly post-transcriptional, including inhibiting FBW7 function. A novel chemical inhibitor of FIR/PUF60 and its splicing variants were revealed to target EMT through FBW7 and E-cadherin in this study. Three-dimensional crystal structure analysis revealed that the U2AF-homology motif (UHM) of FIR or FIRΔexon2 interacted with the WD-like motif in the degron pocket of FBW7. Therefore, the interaction between FIRΔexon2 and FBW7 inhibits FBW7-mediated proteasomal degradation of BRG1 and Snai1. BK697 is a novel, low molecular weight compound containing a WD-like domain targeting FIRΔexon2. Overall, our study suggests that FIRΔexon2-mediated suppression of E-cadherin via the FBW7/BRG1/Snai1 axis prevents EMT and invasion in gastric cancer.

Expectedly, small molecular weight chemicals that interacted with FIRΔexon2 have a WD-like motif and were identified with NPDepo screening^23,40^. Computer screening using the Namiki database (Namiki Shoji Co., Ltd., Tokyo, Japan) was used to search for synthesized chemicals that mimic the structure of the identified compound that consisted of commercially available chemicals. Actually, the chemical skeletons of the two synthesized compounds were regarded as WD-mimicking forms (Fig. 7a and Supplementary Fig. S6)^23^. On the basis of the tests with the synthesized compounds, we modified the chemical structure and finally identified BK697 (Fig. 7a and Supplementary Fig. S6). BK697 suppressed the growth of HeLa cells (Fig. 7b) and affected FIR family, E-cadherin, Snai1, and BRG1 expression (Fig. 7c, d). Notably, the dose of BK697 necessary to increase E-cadherin and SAP155 mRNAs was smaller (50 μM) than that required to increase FIR and FIRΔexon2 mRNAs (100 μM; Fig. 7e). Given the U2AF-homology motif (UHM) of PUF60 directly interacting with the WD-repeat of SAP155 (SF3B1)^13^, FIRΔexon2 is expressed in cancers when *FIR* pre-mRNA is affected by an autocatalytic mechanism through inhibiting the FIR-SAP155 splicing complex^9^. Overexpression of FIRΔexon2 decreased the level of histone acetylation in the *BRG1* genome region but not its mRNAs (Fig. 1c); however, FIRΔexon2 can suppress BRG1 (Fig. 1c, d). Together, FIRΔexon2 suppressed BRG1 post-transcriptional processes (Fig. 8).

## Discussion

Revaluation of co-immunoprecipitated proteins with FIRΔexon2 revealed that FIRΔexon2 engages in post-transcriptional processes with ribosomal proteins, splicing-related factors, mRNA-binding proteins, poly (A) binding proteins, hnRNPs, tRNA, DEAD box and WD-repeat proteins. The transformation/transcription domain-associated protein (TRRAP) that acetylates histones in rDNA transcription was also co-immunoprecipitated with FIRΔexon2 (Fig. 1b)^9^. H3K27ac modification is induced by histone acetyl transferase (HAT), such as GCN5. GCN5 interacts with TRRAP and forms a complex^25^. FIRΔexon2 increases c-Myc expression that activates rDNA transcription^7^ then potentially promotes ribosome synthesis^41^. As expected, FIRΔexon2, but not FIR, acetylated H3K27 on the *BRG1* promoter revealed by ChIP-seq and suppressed BRG1 expression. Further, FIRΔexon2 suppressed BRG1 protein expression, but not BRG1 mRNA expression, indicating that FIRΔexon2 post-transcriptionally regulates BRG1 (Fig. 1c). BRG1 controls multipotent neural crest formation by regulating EMT-related genes with CHD7^1,2^. BRG1 also engages in pre-mRNA splicing by interacting with RNPs in cancers^3–5^. In addition to cancers, *PUF60* and *CHD7* are responsible for the CHARGE syndrome that shows developmental disorders^12^; herein, cooperatively translocating nucleosomes to permit transcription of FGF8 by RNA pol II in neural development. Since siRNA of FIR or FIRΔexon2 surely changed alternative splicing of *FGF8* pre-mRNA (Fig. 2e), FIRΔexon2 possibly cooperates with BRG1 in neural development through alternative splicing of *FGF8* pre-mRNA processing^12^. FGF8 has been reported as a prognostic factor in adenocarcinoma of esophago-gasric junction^42^ and contributes to radiation resistance in rectal cancer^43^. Furthermore, FIRΔexon2 was detected in human gastric cancers but not in mice tumor cells (Fig. 3). The simultaneous increases in BRG1, Snai1, and E-cadherin did not induce EMT in Gan-mice (Fig. 3b, c); however, the expressions of FBW7, BRG1, and E-cadherin decreased in the human gastric cancer tissues (Fig. 4a, b). The expressional change of FIR and FIRΔexon2 increases c-Myc expression that activates rDNA transcription^7^ and then potentially promotes ribosome synthesis^41^. FIRΔexon2 altered ribosomal protein expression by RNA-seq analysis and H3K27ac increased specifically in *FIR/PUF60* genome regions (data not shown) because FIRΔexon2 may directly or indirectly affect the TRRAP complex^9^. These results indicated that FIRΔexon2 possibly relates to rDNA transcription in the nucleolus and to *FIR/PUF60* gene regulation in the nucleoplasm as well. Therefore, autocatalytic production of FIRΔexon2 is crucial for carcinogenesis in terms of ribosome protein synthesis. Together, E-cadherin suppression by FIRΔexon2 at least partly via the FBW7/BRG1/Snai1 axis was shown to promote invasion of gastric cancer cells (Fig. 8).

Given the U2AF-homology motif (UHM) of PUF60 directly interacting with the WD-repeat of SAP155 (SF3B1)^13^, FIRΔexon2 expressed in cancer cells by alternative splicing of FIR pre-mRNA, which is dysregulated by the FIR-SAP155 interaction^9^. FIRΔexon2 transgenic mice were prepared with C57BL/6 mice (UNITECH Co., Ltd., Kashiwa, Chiba, Japan). Body weight curve of FIRΔexon2 transgenic mice was significantly lower than that of wild mice (Supplementary Fig. S7). There was no apparent tumor formation in organs of FIRΔexon2 transgenic mice (data not shown). These results indicated that certain level of sustained FIRΔexon2 expression is required for carcinogenesis. One possible mechanism of persistent FIRΔexon2 activation is the disturbance of FIR pre-mRNA by an autocatalytic mechanism through inhibiting the FIR-SAP155 splicing complex. To test the potential causal relationship between FIRΔexon2 and BRG1 and invasive gastric cancer, an introduction of FIRΔexon2 by CRISPR editing in *FIR*^*+/*^^−^ mouse or PDX (Patient Derived Xenograft) could be expected to induce an invasive tumor in future experiment.

FIR family (FIR, FIRΔexon2, and PUF60), Snai1, and BRG1 increased, but FBW7 and E-cadherin decreased in human gastric cancers, as shown by immunohistochemical staining (Fig. 5a, Supplementary Figs. S4 and S5); however, the expression of BRG1 was reduced in some cases (Fig. 4a and Supplementary Fig. S4). The expression profiles of other proteins were indicated in a mouse-model and in human gastric cancers (Supplementary Figs S2, S4a, b). A wound-healing assay by transfection of E-cadherin-specific siRNA showed enhanced migration of NUGC cells (Fig. 5b). The interaction between UHM of FIR or PUF60 and the WD-like motif (W425 and D399) of FBW7 was examined by three-dimensional crystal structure analysis^17^. The titration curve of FBW7 with FIR∆exon2 suggested a molecular interaction between these two proteins (Fig. 6d). Therefore, the interaction of UHM of FIRΔexon2 could interfere with the WD-like motif of FBW7 and potentially inhibit its function. For clinical applications, BK697 (Fig. S6), a chemical inhibitor of FIRΔexon2, suppressed FIR family expression and tumor cell growth with SAP155 and E-cadherin suppression (Fig. 7c) and increased Snai1 expression (Fig. 7d). All compounds that chemically interact with FIRΔexon2, including BK697, have an aromatic ring connected to a carboxyl group by a short linker. Hence, FIRΔexon2 potentially binds to analogs of the WD-motif of FBW7. Possible interactions between FIRΔexon2 and FBW7 caused the FBW7’s disability to further destabilize Snai1 proteins and resulted in decreased E-cadherin levels that promoted gastric cancers through EMT (Figs. 6 and 7). Therefore, FIRΔexon2 appears to suppress E-cadherin expression at least in part through the FBW7/BRG1/Snai1 axis for promoting EMT and invasion in gastric cancer. The expression of BRG1 was not necessarily increased nor positively correlated with Snai1 in some human cancers (Fig. 4 and Supplementary Fig. S4), which indicated that the FBW7/BRG1/Snai1 axis is perturbed in gastric cancers. Actually, the level of H3K27 histone acetylation in the *BRG1* genome region was found to be decreased by FIRΔexon2 overexpression in vitro (Fig. 1c). Since the expression of cyclin-E is a substrate of FBW7^23^ and E-cadherin is regulated by the FBW7/BRG1/Snai1 pathway^3^, altered or disturbed expression of FIR family possibly affects FBW7 function in gastric cancers. Further study is required to explore alternative pathways in which FIRΔexon2 directly or epigenetically affects *BRG1* gene regulation.

Additionally, *FIR* deficiency promoted alternative splicing to increase pyruvate kinase M2 that engages in the glucose metabolism of cancers in mice thymic lymphoma tissues^44^. Moreover, the long non-coding RNAs (lncRNAs) are a novel class of regulatory genes that have critical roles in cancer progression, and translational regulatory lncRNA (treRNA) downregulates the expression of the epithelial marker E-cadherin by suppressing the translation of its mRNA^45^. A novel mechanism of treRNA is dependent on the 3′UTR of the E-cadherin mRNA, and a novel ribonucleoprotein (RNP) complex, including FIR or PUF60, is required for this treRNA function^45^. The altered FIRΔexon2 expression changes the specific ribonucleoprotein (RNP) complex revealed by RNA-sequencing analysis (data not shown) and, accordingly, FIRΔexon2 potentially generates lncRNAs in cancers.

In conclusion, autocatalytic regulation in alternative splicing of the *FIR/PUF60* gene by the SAP155-FIRΔexon2 complex^9^ simultaneously downregulated FBW7 and E-cadherin via Snai1. This pathway is potentially pivotal for the invasion or metastasis of gastric cancer through EMT. Clinically, BK697 and its derivatives are potential FIRΔexon2 inhibitors and candidate drugs for the treatment of gastric cancer.

## Materials and methods

### Human samples and cell lines

Human gastric cancer tissues were obtained from 14 patients who underwent gastrectomy in the Department of General Surgery, Chiba University Hospital, Chiba, Japan (Supplementary Table S4). Written informed consent was obtained from each patient before surgery after gaining approval by the ethics committee of the Graduate School of Medicine, Chiba University. Fresh tissue samples were frozen immediately in liquid nitrogen and stored at −80 °C until analysis. The definition of clinical stages of gastric cancer patients was determined according to the Japanese Classification of Gastric Carcinoma^46^. Mouse fibroblast NIH3T3 cells, human gastric cancer cell lines MNK7, MNK45, MNK74, NUGC3, and NUGC4 and HeLa (human cervical squamous cell carcinoma cells) cells (purchased from ATCC) were cultured in Iscove’s Modified Dulbecco’s Medium (IMDM) and supplemented with 10% fetal bovine serum (FBS) and 1% penicillin–streptomycin. Cells were grown at 37 °C in a 5% CO_2_ incubator.

### Animal experiments

The fertilized eggs of a Gan-mouse (K19-Wnt1/C2 mE) were obtained from Kumamoto University and matured in Chiba University. We crossbred a male Gan-mouse and female FIR^+/−^ mouse female. The bred FIR^+/−^ mice were maintained in the animal research facility of the Graduate School of Medicine, Chiba University in accordance with institutional guidelines. FIRΔexon2 transgenic mice were prepared with C57BL/6 mice by UNITECH Co., Ltd. (Kashiwa, Chiba, Japan).

### Protein extraction and western blotting

Human frozen tissues (25 mg each) of paired tumor and corresponding adjacent mucosa from the same patients with gastric cancer were pulverized for extraction of proteins. The tissue in the extraction buffer (2 M Thiourea,7 M Urea, 30 mM Tris CL2%, CHAPSProtease inhibitor complete 1 tablet/50 μL, 1% DTT, and 1% Pharmalyte TM 3-1 in dH_2_O) was homogenized three times with homogenizer (Polytron, Tokyo, Japan) for 30 sec to 1 min per homogenization. The homogenate was then centrifuged using an ultra-high speed centrifuge for 1 h at 50 K. The supernatant was collected and stored at −80 °C following measurement of the protein concentration with a Bio-Rad protein assay (Bio-Rad, CA, USA). Western blotting was performed as previously described^9^. The other antibodies used in this study are listed in (Supplementary Table S5).

### Quantitative reverse-transcription-polymerase chain reaction (qRT-PCR)

Total RNA was extracted from cancer cells using a QIAgene Miniprep Kit (Qiagen, Tokyo, Japan). cDNA was synthesized from total RNA using a First Strand cDNA Synthesis Kit for RT-PCR (Roche, Mannheim, Germany). PUF60, FIR and FIRΔexon2 [FIR splicing variants] cDNA were amplified from the cDNA with the respective sets of forward and reverse primer pairs^10,27^ (Fig. 1d, Supplementary Table S6) by using the qRT-PCR reagents (Supplementary Table S7)^9^.

### Chromatin immunoprecipitation (ChIP) and ChIP-qPCR

ChIP assays were performed on approximately 10^7^ cells as previously described^47^. Briefly, the cells were crosslinked with 1% formaldehyde at room temperature for 10 min and formaldehyde was quenched by the addition of 2.5 M glycine to a final concentration of 0.125 M. Crosslinked chromatin was sonicated to a size of 0.2–1 kb using an ultrasonic disruptor [BRANSON Digital Sonifier (BRANSON)]. A total of 1 μg of H3K27ac antibodies and 20 μL of Dynabeads Protein G (Thermo Fisher Scientific Diagnostics) were mixed in IP dilution buffer and incubated at 4 °C overnight. After washing with IP dilution buffer, antibody-binding beads were added to the sonicated-chromatin sample and incubated at 4 °C overnight. The beads were washed and the chromatin was eluted, followed by reversal of crosslinking and DNA purification. Chromatin-immunoprecipitated DNA was dissolved in the elution buffer. Enrichment of ChIP samples were further confirmed with ChIP-qPCR using specifically targeted positive and negative region primer sets (Supplementary Table S6).

### ChIP-seq and data analysis

Libraries were constructed with a KAPA Hyper Prep Kit (Kapa Biosystems) according to the manufacturer’s instructions. ChIP-seq libraries were quantified using a Bioanalyzer (Agilent, USA) and sequenced at a concentration of 6.5 pM on a Hiseq1500 (Illumina, USA) platform or at a concentration of 1.5 pM on a NextSeq500 (Illumina, USA) platform. Sequenced reads in the ChIP-seq experiment were mapped to the UCSC human genome (hg19) using bowtie software. Duplicated reads were removed with Picard tools. Peak calling and motif analysis were performed with HOMER software (http://homer.salk.edu/homer/index.html). HOMER was also used to obtain differential peaks. The H3K27ac peaks in FIR-FLAG or FIRΔexon2-FLAG were compared with untreated HeLa cells.

### Small interfering (si) RNA transfection

E-cadherin siRNA duplexes were purchased from Sigma–Aldrich. Transient transfection of siRNAs was performed using Lipofectamine 2000 (Invitrogen, Japan) according to the manufacturer’s instructions. The transfected cells were cultured at 37 °C for 48 h in a CO_2_ incubator. The target sequences for the siRNAs are listed in (Supplementary Table S7).

### Immunohistochemical staining

Immunohistochemical staining was performed as previously described^5,9^. Human sample list are indicated (Supplementary Tables S4 and S8).

### Wound-healing assay

A wound-healing assay was performed as previously described^9^.

### Stable transfection of pcDNA3.1-FIRΔexon2 plasmids

Stable transfection of pcDNA3.1-FIRΔexon2 plasmids was performed described previously^9^.

### Soft-agar colony formation assay

Soft-agar colony formation assay was performed as previously described^48^. The positive control cells (1 A cells) were kindly provided by Dr. Ariga H^49^.

### Display of the three-dimensional structure of FBW7

The crystal structures, Protein Data Bank (PDB) code: 2QVR^50^ and 2PEH^37^, were visualized with PyMOL (DeLano, W. L.; The PyMOL Molecular Graphics System, Schrödinger, LLC).

### Expression and purification of FIR∆exon2 and FBW7

The *FIR∆exon2* gene was inserted into a pET-50b (+) DNA plasmid vector. An *Escherichia coli* strain, Rosetta (DE3) pLysS (competent cells), that was transformed with the pET-50b-FIR∆exon2 vector, was cultured in 1 L Luria-Bertani (LB) medium at 30°C until the O.D. 600 value reached 0.6. This was followed by 12 h of incubation after an addition of 0.2 mM isopropyl β-D-1 thiogalactopyranoside (IPTG). A cell pellet was obtained by centrifugation of the cultured medium. The pellet was resuspended in a buffer of 50 mM Tris-HCl at pH8.0 and 500 mM NaCl containing 10 mM imidazole and 1 mM phenylmethylsulfonyl fluoride (PMSF). After disrupting the bacterial cell membrane by sonication, the protein was purified with a co-affinity column with a gradient rise of the imidazole concentration. The eluted fraction was dialyzed overnight against the buffer without imidazole. The Nus-tag was cleaved by HRV-3C protease and the protein was purified with a Ni-affinity column to remove the cleaved Nus-tag and HRV-3C protease. The protein was finally purified by gel filtration with a running buffer of 10 mM Tris-HCl at pH 8.0 and 300 mM NaCl. FBW7 was expressed as a complex with Skp-1 using the pCDF-2 plasmid vector. A competent cell, Rosetta (DE3) pLysS, was transformed with the pCDF-2 Nus-tag-fused FBW7-Skp1 vector that was cultured in 1 L of LB medium. After an addition of 0.2 mM IPTG, the competent cells were incubated for 12 h. After resuspension of the cell pellet, the bacterial membrane was disrupted with a French press. Protein purification was performed with a co-affinity column, followed by Nus-tag cleavage with HRV-3C, a Ni-affinity column, and gel filtration.

### Isothermal titration calorimetry (ITC) measurement

The binding affinity between FIR∆exon2 and FBW7 was measured with an isothermal titration calorimetry (ITC) technique using the MicroCal VP-ITC system (Malvern Panalytical, UK). The sample cells were filled with 1400 μL of 50 mM phosphate buffer, pH 7.4, containing 15 μM of the purified Skp1-FWB7 complex. The measurement of binding affinity was performed at 30 °C. The solution of 50 mM phosphate buffer, pH 7.4, containing 300 μM FIR∆exon2 was injected into the sample cells from the syringe for titration. The injection volumes were 10 μL each, the injection time was 20 s, and the injection was 150 s. The titration was repeated 25 times.

### Binding affinity between FIR∆exon2 and FBW7

The titration curve of FBW7 with FIR∆exon2 suggested a molecular interaction between two proteins. The exothermic peaks were observed with the earlier injections. The exothermicity of the injection was decreased in the later injections. The dissociation constant was calculated to be 1.3 × 10^6^ M^−1^ from the ITC thermogram. The binding of FBW7 and FIR∆exon2 was enthalpically favorable because the ITC measurement showed the interaction was exothermic.

### In silico screening for inhibitory compounds against FIRΔexon2 protein function

Small molecular chemical compounds against His-tagged FIR (His-FIR) and His-tagged FIRΔexon2 were screened among 23,275 natural chemicals of NPDepo (RIKEN Natural Products Depository) at RIKEN institutes (Wako, Saitama, Japan) as previously described^51^. In silico computer screening was performed to search fo synthesized chemicals that mimicked the structure of the identified compound using the Namiki database that was composed of commercially available chemicals^23^. Based on the computer screening, 16 compounds were selected and purchased from chemical suppliers (Namiki Shoji Co., Ltd., Tokyo, Japan). Since the WD-motif of SAP155 (SF3B1) directly binds to PUF60^13^, chemical skeletons of the two synthesized compounds contained a WD-like motif (Fig. 7a). Several compounds were selected from the synthesized chemicals^40^. Consequently, we identified a highly active compound, BK697, competing with FIRΔexon2 functions, which suppressed the proliferation of cancer cells^22^.

### Biological effect of BK697, a candidate FIRΔexon2 inhibitor

HeLa and NUGC4 cells were treated with different concentrations of BK697 at different time intervals^22^. Briefly, on day one, NUGC4 or HeLa cells were prepared in IMDM supplemented with 10% FBS. On day two, candidate chemicals that inhibited FIRΔexon2 were diluted in DMSO at a concentration of 10 mM and added as 10 or 20 μL/well/2 mL in the medium (the final concentration in the medium was 50 and 100 μM, respectively) or added as 20 or 60 μL/well/2 mL medium (the final concentration in the medium was 100 and 300 μM, respectively). A total of 100 or 300 μM of BK697 was exposed to NUGC4 cells for 24 h, and 50 or 100 μM of BK697 was exposed to NUGC4 cells or HeLa cells for 6 h, 24 h and 48 h at 37 °C in a CO_2_ incubator.

### Statistical analysis

Statistical significance of the differences in numerical data was assessed with a Student’s *t*-test and the Wilcoxon test. All tests were two-tailed and a *P*-value below 0.05 was considered significant.

## Supplementary information

Supplemental Figure and Table legends

Supplemental Figure1

Supplemental Figure2

Supplemental Figure3

Supplemental Figure4

Supplemental Figure5

Supplemental Figure6

Supplemental Figure7

Supplemental Table1

Supplemental Table2

Supplemental Table3

Supplemental Table4

Supplemental Table5

Supplemental Table6

Supplemental Table7

Supplemental Table8
