# Supplementary material for: Post-transcriptional regulation of BRG1 by FIRΔexon2 in gastric cancer
Source: Oncogenesis. 2020 Feb 18;9(2):26. doi: 10.1038/s41389-020-0205-4 (PMC7028737; doi:10.1038/s41389-020-0205-4)
Supplement: Supplementary file 1 — Supplemental Figure and Table legends [file 41389_2020_205_MOESM1_ESM.docx]

**Supporting Information**

Table S1. List of commonly immunoprecipitated proteins with FIR or FIRΔexon2 detected by a nanoflow liquid chromatography-tandem mass spectrometry.

Table S2. Characteristics of the mice used in the experiment.

Table S3. Protein expression profiles in Gan-mouse and human gastric tumor tissue.

Table S4. List of gastric cancer patients used in this study.

Table S5. Primary and secondary antibodies for western blot and immunohistocemical staining.

Table S6. Primers and qRT-PCR conditions used in this study.

Table S7. Primers probes, PCR conditions for qRT-PCR, CHIP-qPCR and sequences for siRNAs used in this study.

Table S8. List of gastric cancer patients for immunohistochemical staining.

**Figure S1. Five gastric cancer cell lines, MNK7, MNK45, MNK74, NUGC3, and NIGC4, were examined their FGF8 expression at protein and mRNA levels.** (**a**) NUGC4 indicated highest expression of FGF8 expression among five gastric cancer cell lines, MNK7, MNK45, MNK74, NUGC3, and NIGC4. (**b**) FGF8 mRNA was detected by qRT-PCR in those cells. HPRT, hypoxanthine phosphoribosyltransferase, was used as an internal control. (**c**) NUGC4 showed morphologically round cell shape.

**Figure S2. Protein expression profiles of mouse gastric tumor tissue.** (**a**) Proteins’ expression profiles in human gastric cancer tissues. The extent of the signals detected by Western blot was quantified by densitometry analysis. (**b**) *FIR^+/−^* affected the expressions of c-Myc, cyclin-E, and Snai1 but not the expressions of E-cadherin and FBW7. Remarkably, the expressions between BRG1 and Snai1 was significantly correlated (R^2^ = 0.973). The expressions between Snai1and E-cadherin showed weak positive correlation (R^2^ = 0.263). The expression of SAP155 was significantly correlated with the expression of a splicing factor heterogeneous nuclear ribonucleoprotein A1) (hnRNPA1). Correlation of protein expression of mouse gastric tumor tissue determined by statistical tests. N = 12, R < 1.0 obtained by Student’s t-test. Protein expression changes according to the tumor size of mouse gastric tumors. Protein expression was determined by statistical processing. N = 12, P < 0.01 was considered statistically significant by Student’s t-test. Three different genotypes of mice are indicated as follows,


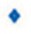
Wild-type mouse (Figure 1, lanes 1–3),


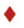
Gan mice (gastric tumor wet weight less than 0.5 g) (Figure 1, lanes 4–6),


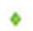
Gan mice (gastric tumor weight more than 2.0g) (Figure 1, lanes 7–9)

*
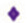
FIR^+/−^* mice (Figure 1, lanes 10–12).

**Figure S3.** **Expression of FIRs and related proteins in human gastric cancer tissues examined by western blotting.** Expression of FIR and related proteins were examined by western blotting in nine paired tumor (T) and adjacent non-tumor (N) tissue samples from human gastric cancer patients. Gender/age, pathology and tumor stages are listed above. FIRs, Snai1, BRG1, E-cadherin, FBW7, c-Myc, and SAP155 were examined by western blot. β-actin was used as internal control.

**Figure S4. Protein expression profiles of human gastric cancer tissue samples. (a)** The extent of the signals detected by western blotting was quantified by densitometry indicated in Figure2. T: cancer, N: non-cancer tissues. **(b)** The expressions of BRG1 and Snai1 were not significantly correlated in human gastric cancer tissues. The expressions of FBW7 and cyclin-E were negatively correlated (R^2^ = 0.353), but the expressions of Snai1 and E-cadherin were not correlated (R^2^ = 0.199) in human gastric cancers (T) and non-cancerous tissues (N). There was a slight negative correlation between Snai1 and E-cadherin in human invasive gastric cancers. Correlation of proteins’ expression of T and N determined by statistical analysis. FIRs expression was positively correlated with cyclin-E expression in human gastric cancer tissues as observed in the Gan and *FIR^+/−^* mice. The expression of BRG1 was not correlated with Snai1 expression in human gastric cancers (R^2^ = 0.04), which was different from mice gastric tumors. **(c)** Snai1 expression was increased but BRG1 was decreased in (T) than those in (N). The thin-gray bar represents mice samples. The dark-gray bar indicates human samples. The expression ratio of T/N=1 is indicated as horizontal line. The T/N ratios of the expressions of FBW7, E-cadherin, and BRG1 were ≤1 in human invasive gastric cancer tissues (n = 5) but >1 in gastric tumors of Gan mice (n = 6).

**Figure S5. Expression of FIR family, and FBW7 and E-cadherin in gastric cancer tissues.** Expressions of E-cadherin, FBW7 and FIRs in gastric cancer tissues were examined by immune-histochemical staining. In non-invasive early stages (IA) of differentiated cancers, the expression of E-cadherin and FBW7 were decreased. On the other hand, in some invasive poorly differentiated gastric cancers, FIRs and FBW7 were increased. In non-invasive early stages (IA) of differentiated cancers, expression of E-cadherin and FBW7 were decreased compared to the poorly differentiated cancers (cf. Table S3).

**Figure S6. Candidates of chemical structural formula that inhibit FIRΔexon2 protein function by in silico computer screening.** Synthesized compounds intended for the inhibitor of FIRΔexon2 protein function. All of the compounds bear a chemical skeleton of aromatic ring connected to carboxyl group with a short linker. Hence, these compounds are analogues of WD motif of FBW7.

**Figure S7. FIRΔexon2 transgenic mice showed significantly lower body weight curve than that of wild mice without apparent tumors.** FIRΔexon2 transgenic mice were prepared with C57BL/6 mice (UNITECH Co., Ltd., Kashiwa, Chiba, Japan). FIRΔexon2 transgenic mice showed significantly lower body weight curve than that of wild mice without apparent tumors, in male (**a**) and female mice (**b**), indicating certain level of sustained FIRΔexon2 expression needs tumorigenesis. Statistical analyses was performed by Student’s t-test. (**c**) Plasmid construct of transgenic mice carrying human FIRΔexon2 cDNA as ORF introduced into C57BL/6. Primers and PCR product sized were indicated in pCMV-TRE/ORF/polyA and pCMV/tTA/polyA construct prepared by UNITECH Co., Ltd. (Kashiwa, Chiba, Japan).
