# Supplementary figures and images for: Post-transcriptional regulation of BRG1 by FIRΔexon2 in gastric cancer
Source: Oncogenesis. 2020 Feb 18;9(2):26. doi: 10.1038/s41389-020-0205-4 (PMC7028737; doi:10.1038/s41389-020-0205-4)

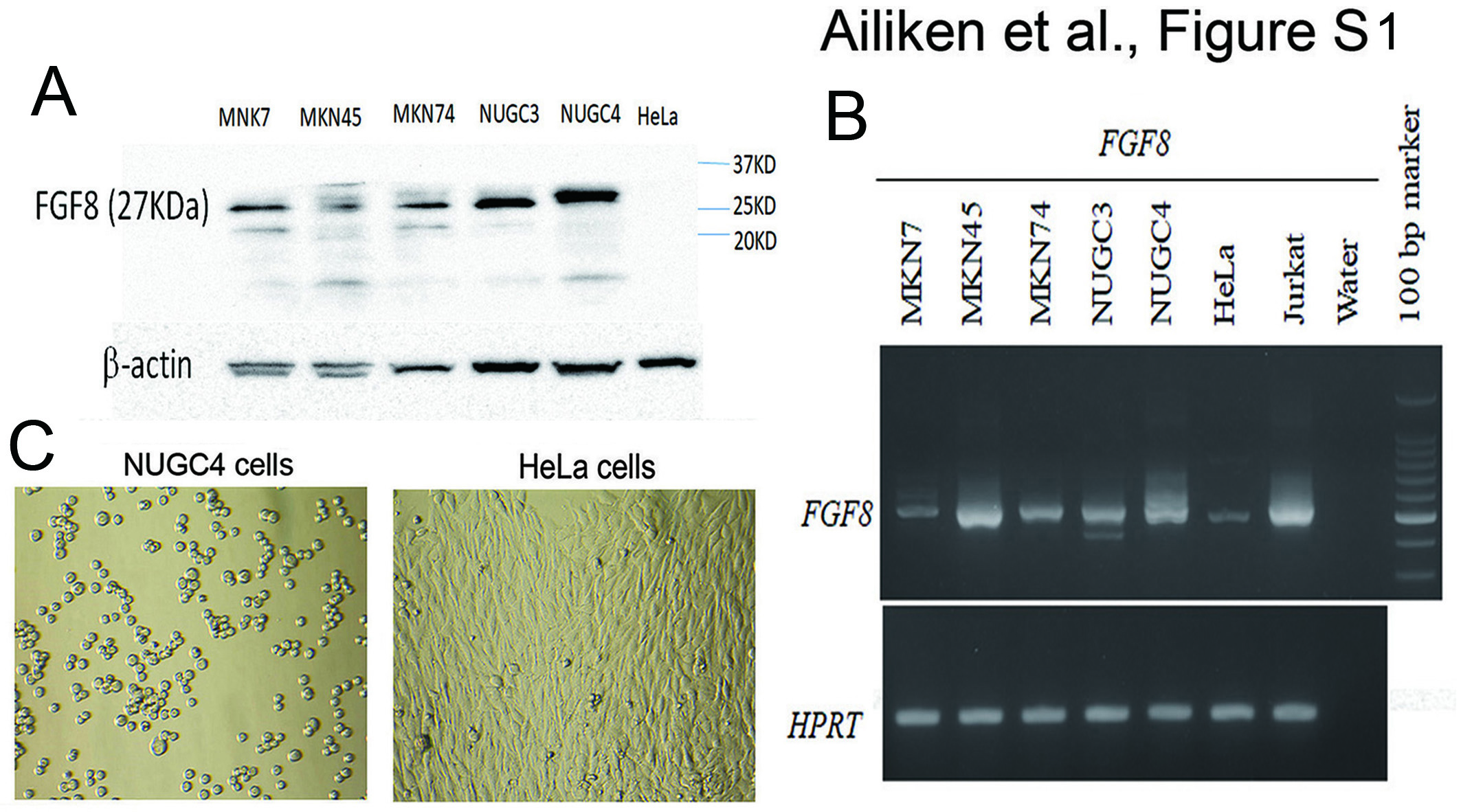

Supplement: Supplementary file 2 — Supplemental Figure1 [file 41389_2020_205_MOESM2_ESM.jpg]

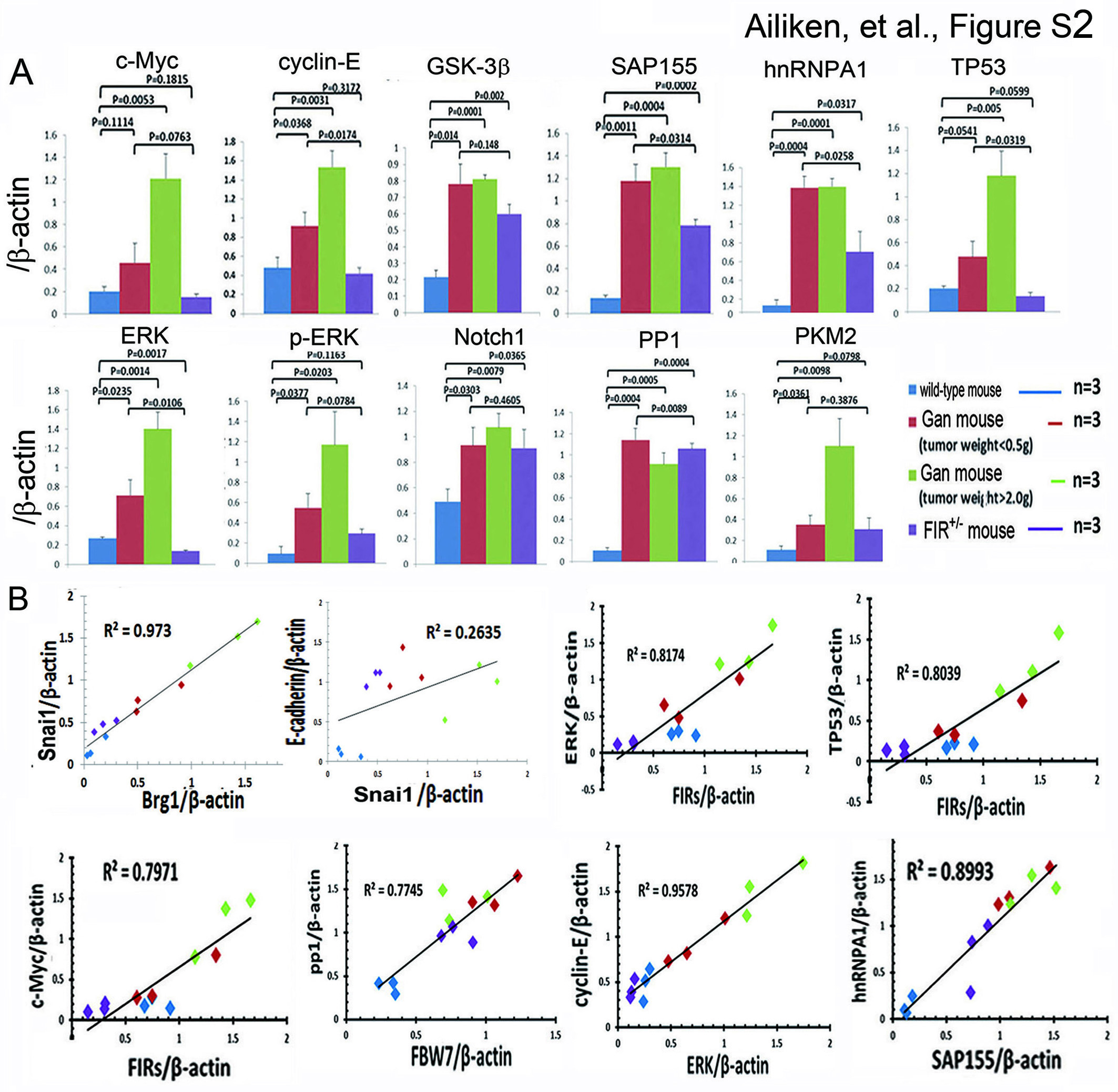

Supplement: Supplementary file 3 — Supplemental Figure2 [file 41389_2020_205_MOESM3_ESM.jpg]

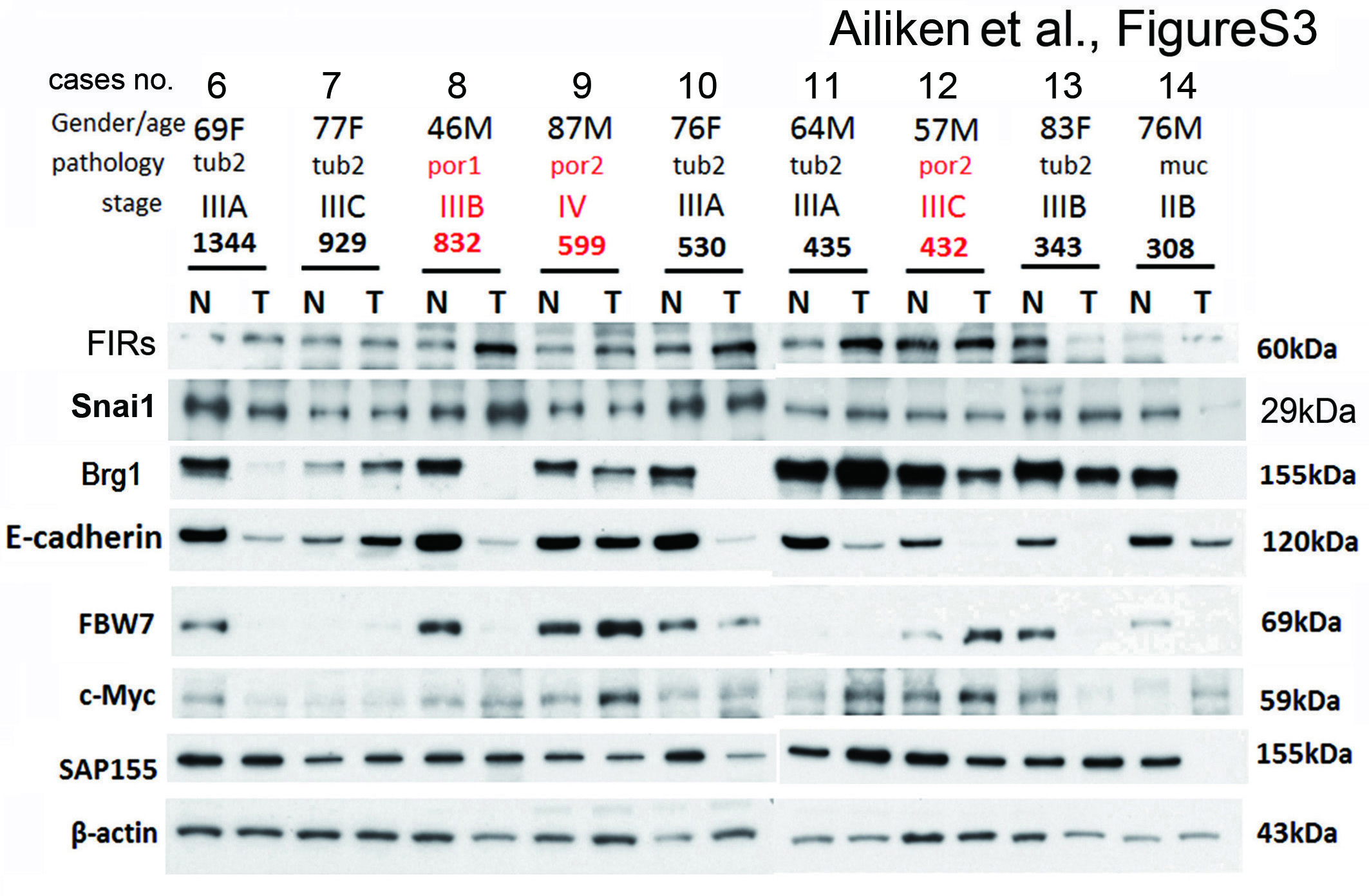

Supplement: Supplementary file 4 — Supplemental Figure3 [file 41389_2020_205_MOESM4_ESM.jpg]

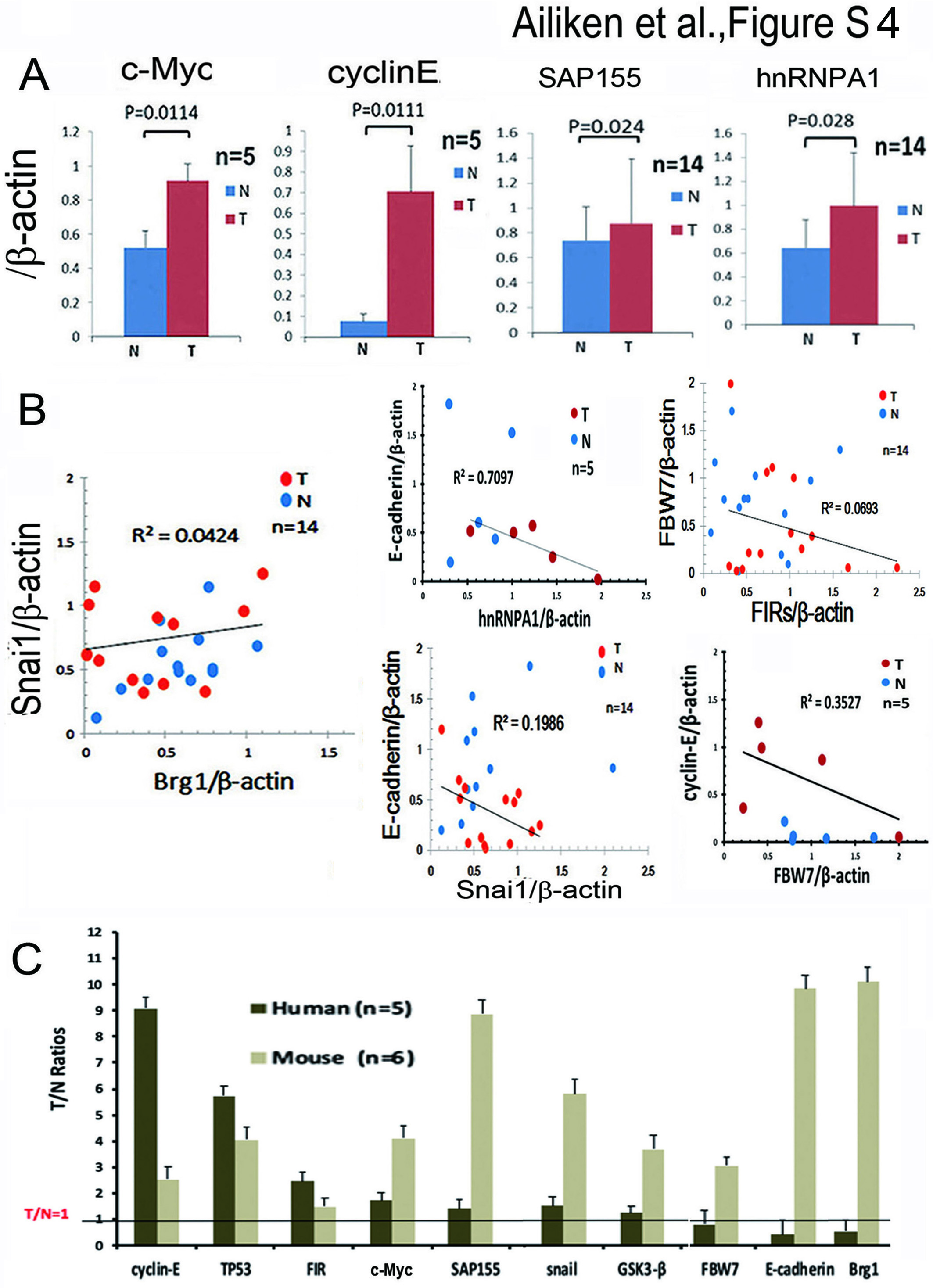

Supplement: Supplementary file 5 — Supplemental Figure4 [file 41389_2020_205_MOESM5_ESM.jpg]

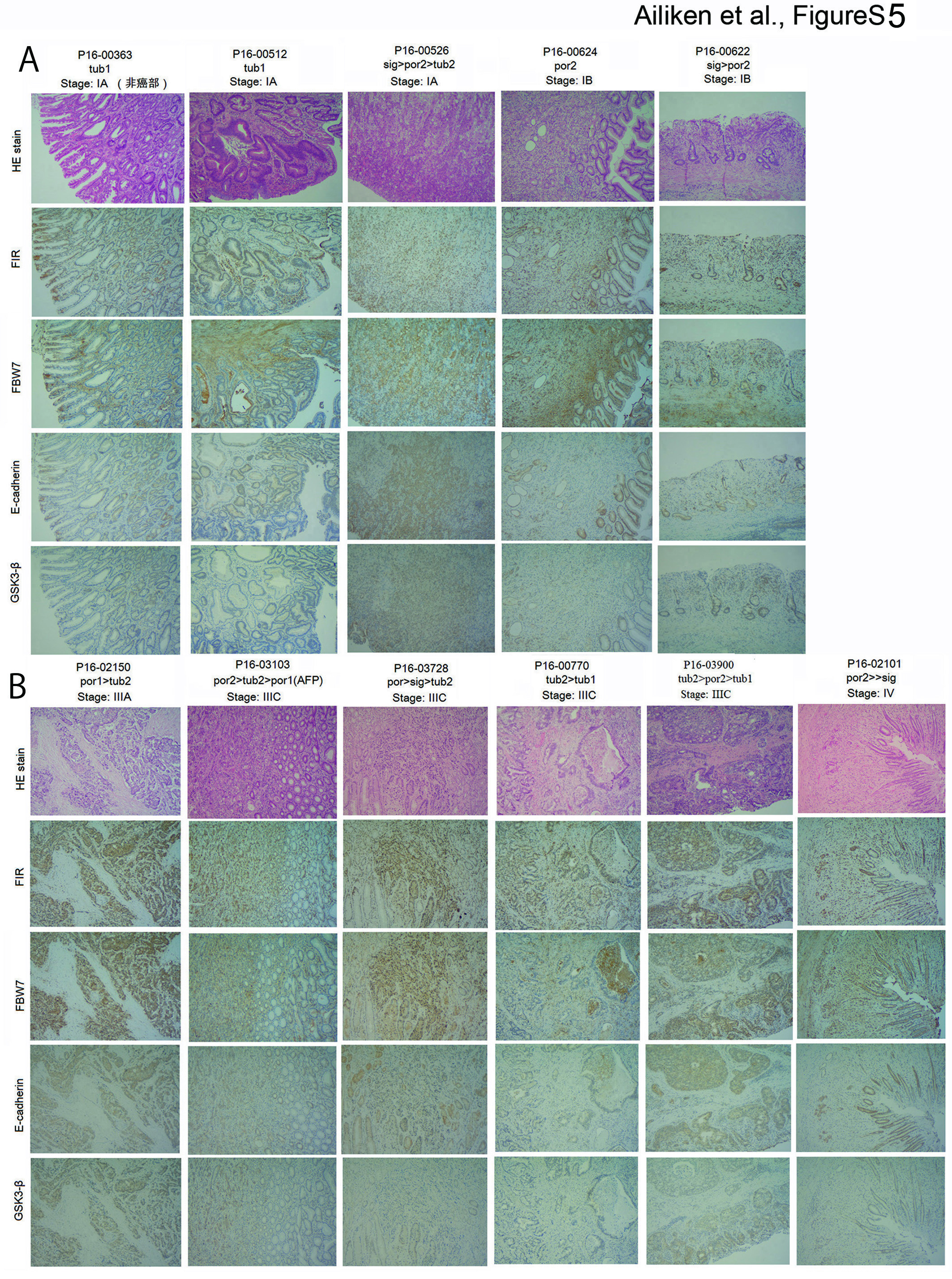

Supplement: Supplementary file 6 — Supplemental Figure5 [file 41389_2020_205_MOESM6_ESM.jpg]

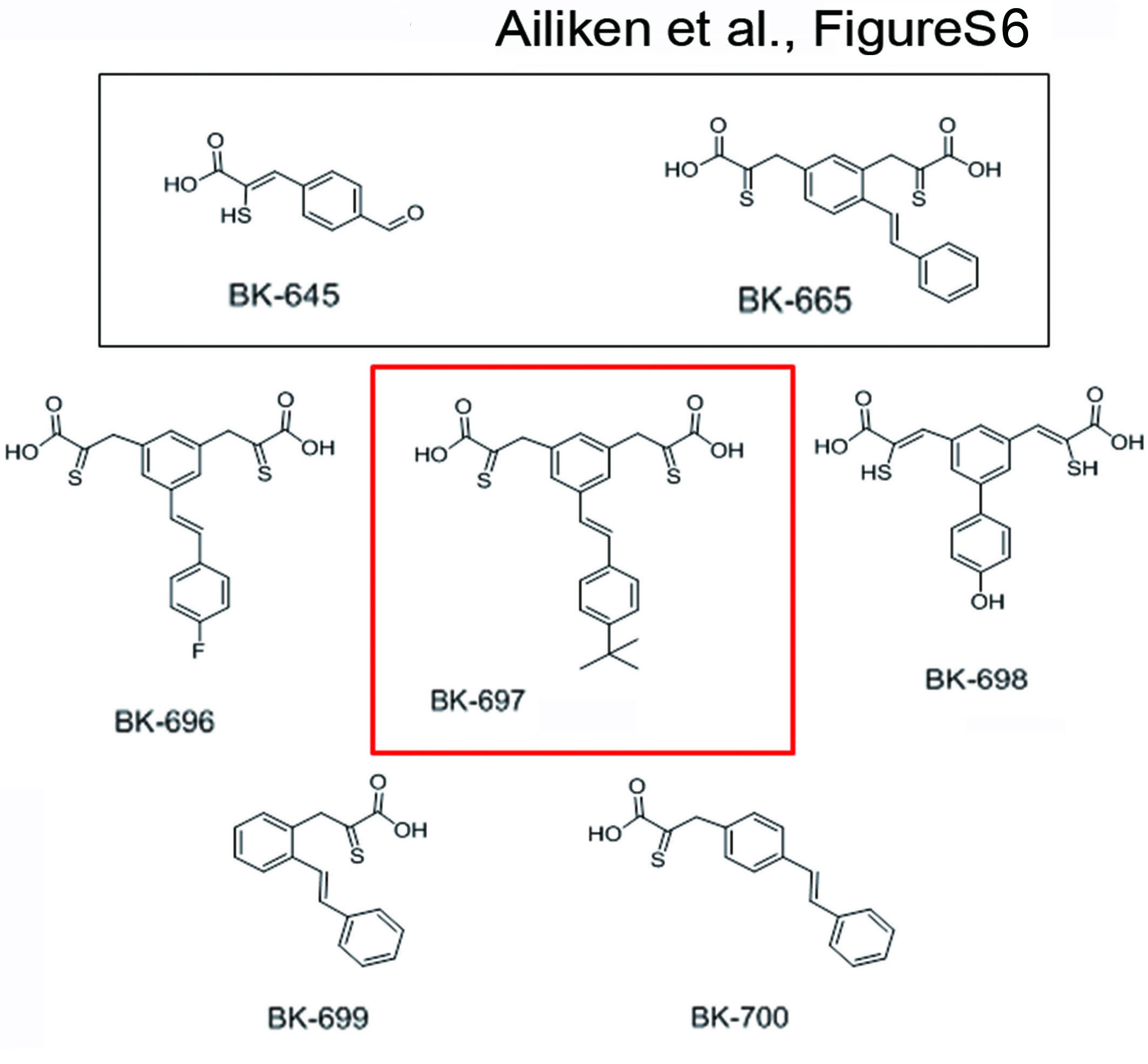

Supplement: Supplementary file 7 — Supplemental Figure6 [file 41389_2020_205_MOESM7_ESM.jpg]

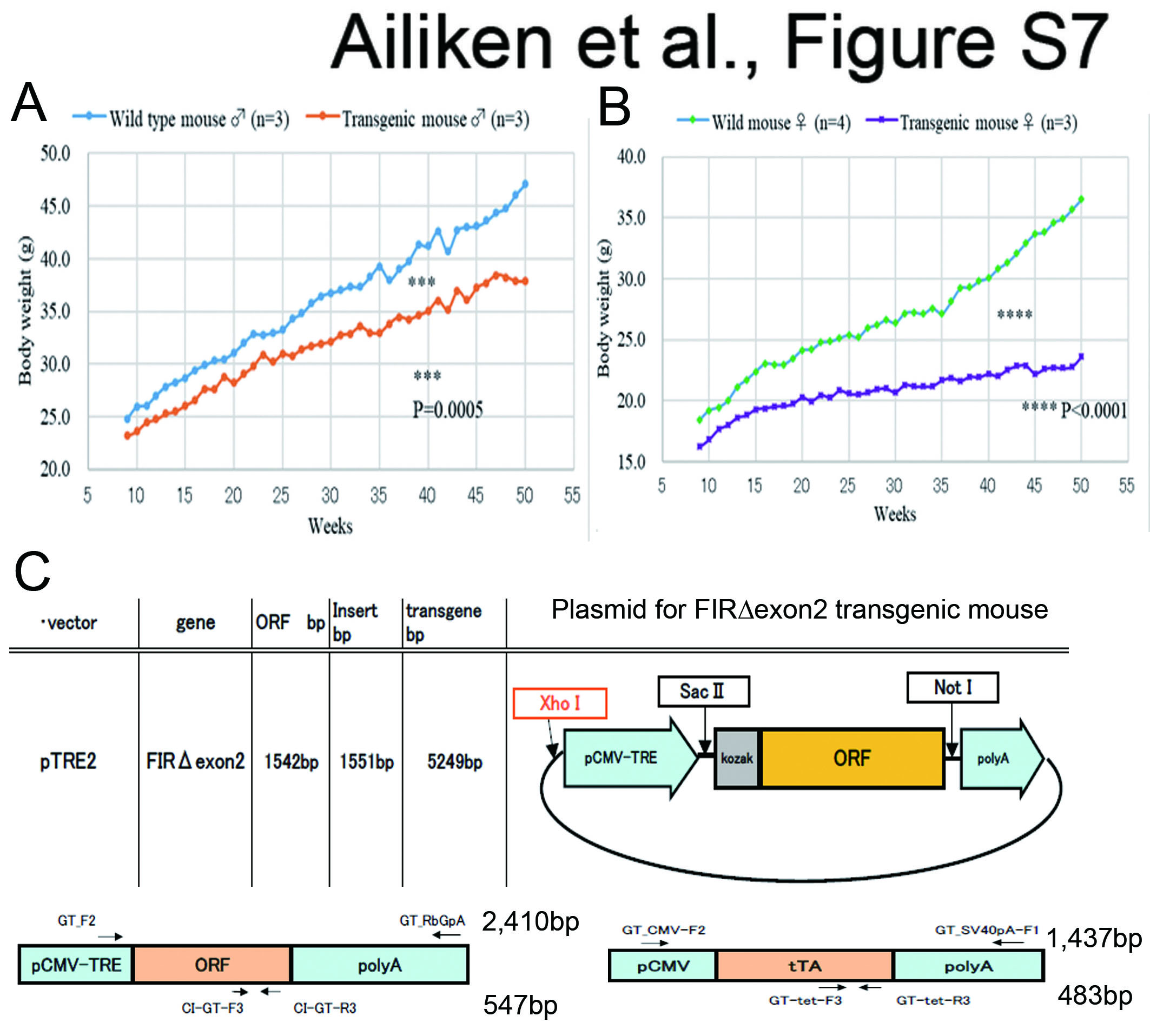

Supplement: Supplementary file 8 — Supplemental Figure7 [file 41389_2020_205_MOESM8_ESM.jpg]
