# Supplementary material for: Post-transcriptional regulation of BRG1 by FIRΔexon2 in gastric cancer
Source: Oncogenesis. 2020 Feb 18;9(2):26. doi: 10.1038/s41389-020-0205-4 (PMC7028737; doi:10.1038/s41389-020-0205-4)
Supplement: Supplementary file 9 — Supplemental Table1 [file 41389_2020_205_MOESM9_ESM.pdf]

TableS1. List of commonly immunoprecipitated proteins FIR and FIRΔexon2 detected by a direct nanoflow liquid chromatography-tandem mass spectrometry analysis

1	DEAD (Asp-Glu-Ala-Asp) box polypeptide 5
2	far upstream element-binding protein family
3	heterogeneous nuclear ribonucleoprotein A1
4	heterogeneous nuclear ribonucleoprotein; A1 or A1-like
5	nuclear ribonucleoprotein A1 family
6	heterogeneous nuclear ribonucleoprotein A2/B1
7	heterogeneous nuclear ribonucleoprotein A3
8	heterogeneous nuclear ribonucleoprotein A/B; isoform a
9	heterogeneous nuclear ribonucleoprotein; A/B or D
10	heterogeneous nuclear ribonucleoprotein D; isoform c
11	heterogeneous nuclear ribonucleoprotein K
12	heterogeneous nuclear ribonucleoprotein L
13	heterogeneous nuclear ribonucleoprotein M; isoform a
14	heterogeneous nuclear ribonucleoprotein R
15	heterogeneous nuclear ribonucleoprotein U-like 1
16	heterogeneous nuclear ribonucleoprotein D-like
17	poly(A) binding protein, cytoplasmic 1 or poly(A) binding protein, cytoplasmic 3
18	poly(A) binding protein, cytoplasmic 1
19	poly(A) binding protein, cytoplasmic 1 or poly(A) -binding protein, cytoplasmic 1-like or poly(A) binding protein, cytoplasmic 5
20	poly(A) binding protein, cytoplasmic 1 or poly A binding protein, cytoplasmic 4
21	poly(A) binding protein, cytoplasmic 1-like 2B or poly A binding protein, cytoplasmic 4
22	poly A binding protein, cytoplasmic 4
23	poly(A) binding protein, nuclear 1
24	poly(rC) binding protein; 1 or 2 or 3 or 4
25	polypyrimidine tract-binding protein 1; isoform d
26	splicing factor 3b, subunit 1(SAP155)
27	splicing factor 3B, 14 kDa subunit
28	splicing factor 3b, subunit 3(SAP130)
29	splicing factor 3b, subunit 4
30	splicing factor proline/glutamine rich (polypyrimidine tract binding
