# Supplementary material for: Post-transcriptional regulation of BRG1 by FIRΔexon2 in gastric cancer
Source: Oncogenesis. 2020 Feb 18;9(2):26. doi: 10.1038/s41389-020-0205-4 (PMC7028737; doi:10.1038/s41389-020-0205-4)
Supplement: Supplementary file 11 — Supplemental Table3 [file 41389_2020_205_MOESM11_ESM.pdf]

Table S3. Protein expression profiles in Gan-mouse and human gastric tumor tissue.				
	**Human (n=5)**		**Mouse (n=6)**	
**protein**	**stomach tumor(T)/ non cancer (N) (n=5)**	**expression**	**non cancer (N) (n=6)**	**expression**
cyclin-E	9.09	over expression	2.54	over expression
TP53	5.74	over expression	4.06	over expression
FIR	2.46	over expression	1.48	over expression
PP1	2.18	over expression	3.67	over expression
hnRNPA1	2.04	over expression	10.19	over expression
PKM2	1.77	over expression	6.52	over expression
c-myc	1.74	over expression	4.11	over expression
SAP155	1.42	over expression	8.88	over expression
ERK	1.35	over expression	3.97	over expression
Snai1	1.53	over expression	5.82	over expression
GSK3-β	1.25	over expression	3.7	over expression
**Brg1**	0.54	**low expression**	10.1	over expression
**FBW7**	0.81	**low expression**	3.06	over expression
**E-cadherin**	0.41	**low expression**	9.84	over expression
**p-ERK**	0.17	**low expression**	9.05	over expression
