# Supplementary material for: Post-transcriptional regulation of BRG1 by FIRΔexon2 in gastric cancer
Source: Oncogenesis. 2020 Feb 18;9(2):26. doi: 10.1038/s41389-020-0205-4 (PMC7028737; doi:10.1038/s41389-020-0205-4)
Supplement: Supplementary file 16 — Supplemental Table8 [file 41389_2020_205_MOESM16_ESM.pdf]

Table S8. List of gastric cancer patients for immunohistochemical staining.

sample number	sex	pathology type	clinical stages
P12-00361	M	tub1>tub2>sig	ⅠA
P12-00363	F	por1>por2>tub2>tub1	ⅠA
P12-00526	M	tub2>tub1	ⅠA
P12-00770	F	tub2>tub1>pap>por2	ⅢC
P12-03103	F	muc>sig	ⅢC
P16-00624	F	tub2>tub1>por2>sig	ⅠB
P16-00622	M	tub2>por2	ⅠB
P16-00512	M	por1>tub2>pap	ⅠA
P16-02101	M	por2>por1>tub2>tub1	Ⅳ
P16-03900	M	tub2>por1	ⅢC
P16-02150	M	tub2>por2>muc	ⅢA
P16-03728	M	por2>sig>muc	ⅢC

pap: papillary adenocarcinoma
tub1: well differentiated tubular adenocarcinoma,
tub2: moderately differentiated tubular adenocarcinoma,
por1: poorly differentiated adenocarcinoma, solid type,
por2: poorly differentiated adenocarcinoma, non-solid type,
sig: signet-ring cell carcinoma
muc: mucinous adenocarcinoma.
